# Effect of High- versus Low-Intensity Supervised Aerobic and Resistance Training on Modifiable Cardiovascular Risk Factors in Type 2 Diabetes; The Italian Diabetes and Exercise Study (IDES)

**DOI:** 10.1371/journal.pone.0049297

**Published:** 2012-11-21

**Authors:** Stefano Balducci, Silvano Zanuso, Patrizia Cardelli, Laura Salvi, Alessandra Bazuro, Luca Pugliese, Carla Maccora, Carla Iacobini, Francesco G. Conti, Antonio Nicolucci, Giuseppe Pugliese, for the Italian Diabetes Exercise Study (IDES) Investigators

**Affiliations:** 1 Department of Clinical and Molecular Medicine, “La Sapienza” University, Rome, Italy; 2 Diabetes Unit, Sant’Andrea Hospital, Rome, Italy; 3 Metabolic Fitness Association, Monterotondo, Rome, Italy; 4 School of Science, University of Greenwich, London, United Kingdom; 5 Laboratory of Clinical Chemistry, Sant’Andrea Hospital, Rome, Italy; 6 Department of Clinical Pharmacology and Epidemiology, Consorzio Mario Negri Sud, S. Maria Imbaro, Chieti, Italy; University of Bath, United Kingdom

## Abstract

**Background:**

While current recommendations on exercise type and volume have strong experimental bases, there is no clear evidence from large-sized studies indicating whether increasing training intensity provides additional benefits to subjects with type 2 diabetes.

**Objective:**

To compare the effects of moderate-to-high intensity (HI) versus low-to-moderate intensity (LI) training of equal energy cost, i.e. exercise volume, on modifiable cardiovascular risk factors.

**Design:**

Pre-specified sub-analysis of the Italian Diabetes and Exercise Study (IDES), a randomized multicenter prospective trial comparing a supervised exercise intervention with standard care for 12 months (2005–2006).

**Setting:**

Twenty-two outpatient diabetes clinics across Italy.

**Patients:**

Sedentary patients with type 2 diabetes assigned to twice-a-week supervised progressive aerobic and resistance training plus exercise counseling (n = 303).

**Interventions:**

Subjects were randomized by center to LI (n = 142, 136 completed) or HI (n = 161, 152 completed) progressive aerobic and resistance training, i.e. at 55% or 70% of predicted maximal oxygen consumption and at 60% or 80% of predicted 1-Repetition Maximum, respectively, of equal volume.

**Main Outcome Measure(s):**

Hemoglobin (Hb) A_1c_ and other cardiovascular risk factors; 10-year coronary heart disease (CHD) risk scores.

**Results:**

Volume of physical activity, both supervised and non-supervised, was similar in LI and HI participants. Compared with LI training, HI training produced only clinically marginal, though statistically significant, improvements in HbA_1c_ (mean difference −0.17% [95% confidence interval −0.44,0.10], P = 0.03), triglycerides (−0.12 mmol/l [−0.34,0.10], P = 0.02) and total cholesterol (−0.24 mmol/l [−0.46, −0.01], P = 0.04), but not in other risk factors and CHD risk scores. However, intensity was not an independent predictor of reduction of any of these parameters. Adverse event rate was similar in HI and LI subjects.

**Conclusions:**

Data from the large IDES cohort indicate that, in low-fitness individuals such as sedentary subjects with type 2 diabetes, increasing exercise intensity is not harmful, but does not provide additional benefits on cardiovascular risk factors.

**Trial Registration:**

www.ISRCTN.org ISRCTN-04252749.

## Introduction

A large body of evidence indicates that physical activity (PA) is associated with reduced cardiovascular disease (CVD) and all-cause mortality in the general population (1,2) and in subjects with type 2 diabetes (3,4). More recently, planned or structured PA, i.e. exercise training, was found to produce additional benefits beyond those of PA itself in individuals with type 2 diabetes (5). In fact, meta-analyses of small-sized studies showed that supervised exercise is effective in improving cardio-respiratory fitness (6) as well as glycemic control and other CVD risk factors (7,8). Moreover, the large Italian Diabetes and Exercise Study (IDES) demonstrated that twice-weekly supervised, facility-based, aerobic and resistance training on top of exercise counseling is superior to counseling alone in promoting PA, improving physical fitness, hemoglobin (Hb) A_1c_ and CVD risk profile, and reducing medication number and/or dosage in sedentary patients with type 2 diabetes (9). The incremental benefits of supervised exercise were largely explained by the achievement of a higher volume of PA, well above the currently recommended amount (10). Combined aerobic and resistance exercise was reported to be more effective than either one alone on glycemic control in two other large trials (11,12), though a systematic review and meta-analysis including these studies (13) showed that structured exercise training, either aerobic, resistance, or both, is associated with HbA_1c_ reduction in patients with type 2 diabetes, especially if of more than 150 minutes per week and when combined with dietary advice.

Based on this evidence, the joint position statement of the American College of Sports Medicine (ACSM) and the American Diabetes Association (ADA) (14) recommends that individuals with type 2 diabetes perform at least 150 minutes/week of moderate-to-vigorous aerobic exercise, plus moderate-to-vigorous resistance training at least 2–3 days/week. In addition, the ACSM/ADA position statement recommends to train under the supervision of exercise professionals and encourages to increase total daily unstructured (commuting, occupational, home and leisure-time) PA. However, while indications of type and volume of PA/exercise have strong experimental grounds, recommendations concerning intensity are based on a meta-analysis including of nine structured aerobic exercise (not resistance) intervention studies of small size (8–21 subjects in the intervention group; n = 132 total) and duration ranging from 8 to 52 weeks (15).

Thus, there is no evidence from randomized controlled trials of adequate size that moderate-to-high intensity (HI) training provides more benefits than low-to-moderate intensity (LI) training on glycemic control and other modifiable CVD risk factors in individuals with type 2 diabetes. A recent, small-sized study showed that continuous LI aerobic exercise is as effective as HI aerobic exercise in lowering HbA_1c_ in obese patients with type 2 diabetes (16), and previous exercise intervention trials in healthy and/or glucose-intolerant individuals reported that HI training is less (17), equally (18,19) or more (20) effective than LI training in improving insulin sensitivity. Furthermore, unsupervised exercise of different durations and intensities in sedentary, overweight women produced similar effects on weight loss and cardio-respiratory fitness (21). Conversely, in one of the studies showing no benefit from HI versus LI exercise on insulin resistance (19), improvements in cardio-respiratory fitness and CVD risk factors were more marked with HI than with LI training (22). More importantly, most of the studies addressing this issue are flawed by the higher volume of work, i.e. energy expenditure, in subjects exercising at a higher intensity, as outlined by Blair et al (23).

This study was aimed at verifying, in the large IDES cohort, the hypothesis that training at HI is more effective than LI exercise of equal energy cost, in improving modifiable CVD risk factors in subjects with type 2 diabetes engaged in a supervised mixed (aerobic and resistance) exercise program.

## Materials and Methods

The protocol of this multicenter randomized controlled trial and CONSORT checklist are available as supporting information (see IDES Protocol S2 and CONSORT checklist S3). The research protocol was approved by the locally appointed ethics committees and participants gave written informed consent.

### Setting and Participants

The IDES enrolled 22 outpatient diabetes clinics throughout Italy between October 1^st^, 2005 and March 31^st^, 2006 (9,24). Each center was connected with a Metabolic Fitness Center, a dedicated facility where patients trained under the supervision of an exercise professional. Sedentary Caucasian patients with type 2 diabetes, according to the ADA definition (25), fulfilling the International Diabetes Federation (IDF) criteria for the metabolic syndrome (26), were eligible for this study, whereas subjects having any condition limiting or contraindicating PA were excluded. Of the 691 eligible patients, 85 were excluded for various reasons (9) and 606 were recruited and randomized to supervised training plus structured exercise counseling (exercise, EXE, group; n = 303) versus counseling alone as part of standard care (control, CON, group; n = 303) for 12 months. Randomization was stratified by center and, within each center, by age (<60 versus ≥60 years) and type of diabetes treatment (no insulin versus insulin, either alone or in combination with oral agents), using a permuted-block randomization software.

**Figure 1:**
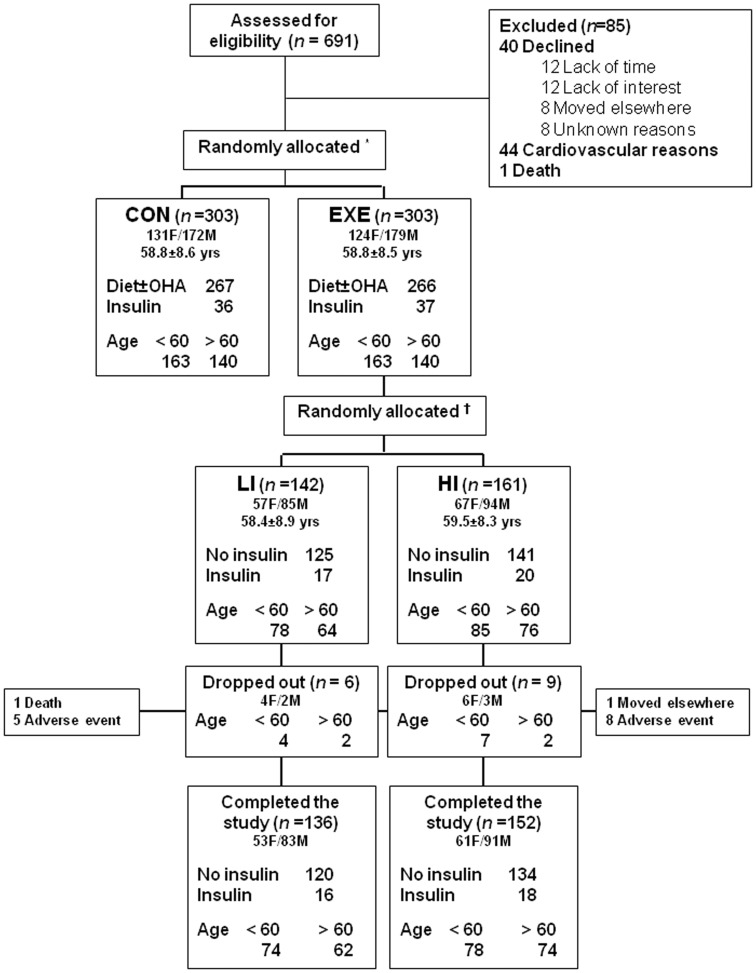
Study flow diagram. * Patients were randomly allocated by center and, within each center, by age (<60 versus ≥60 years) and type of diabetes treatment (no insulin versus insulin) to CON or EXE group and then † EXE participants were further randomized to LI or HI subgroup by center only. CON = control group; EXE = exercise group; LI = low intensity; HI = high intensity.

Participants in the EXE group were further allocated to either LI (n = 142) versus HI (n = 161) training within the intensity range indicated by the guidelines of the time (27). Allocation was made according to the center, which was preliminary assigned to train patients at either LI or HI. The final number of patients in the two subgroups resulted from the lower enrolment rate, on average, in LI than in HI centers. The study flow-chart is reported in Figure 1. Physicians and patients were not blinded to randomization to EXE or CON group, but assignment to LI or HI subgroup was kept hidden to the EXE subjects and sample blinding at central laboratory was achieved using bar codes.

### Interventions

The supervised training program for the exercise group consisted of twice a week supervised sessions of mixed (aerobic and resistance) exercise, based on recent evidence (11,12) and guidelines (10,14) recommending both types of exercise.

Aerobic training was performed using treadmill, step, elliptical, arm or cycle-ergometer. Resistance training consisted of 4 resistance exercises, i.e. thrust movement on the transverse plane (chest press or equivalent), traction movement on the frontal plane (lateral pull down or equivalent), squat movement (leg press or equivalent), and trunk flexion for the abdominals, plus three stretching positions (9,24).

Individuals in the low-to-moderate intensity (LI) exercise subgroup performed aerobic training at 55% of predicted maximal oxygen consumption (VO_2max_) and resistance training at 60% of predicted 1-Repetition Maximum (1-RM), i.e. the maximum amount of weight one can lift in a single repetition for a given exercise. Subjects in the moderate-to-high intensity (HI) exercise subgroup performed aerobic training at 70% of predicted VO_2max_ and resistance training at 60% of predicted 1-RM (see below for methods of estimation of VO_2max_ and 1-RM).

Duration of aerobic training and number of series of resistance training were varied to obtain the same caloric expenditure per kg body weight in the two subgroups, independent of intensity. Intensity was adjusted according to improvements in predicted VO_2max_ and 1-RM, as assessed monthly throughout the study. In addition, caloric expenditure was increased progressively by 0.1 kcal/kg body weight/session every month from 3.0 to 4.1 kcal/kg body weight/session (9,24). More in detail, in order to achieve the pre-defined exercise intensity and volume, the exercise professionals supervising the training sessions calculated every month the aerobic and resistance workload for each machine and the duration or number of repetitions, from the patient’s actual body weight and predicted VO_2max_ or 1-RM, respectively, and the monthly goal of caloric expenditure per session. Then, they set the machines accordingly and ensured that patients completed the training program as established. In addition, for aerobic training, all exercise data were verified at the end of the session as recorded by each machine onto an USB flash drive by the use of the Wellness System TGS (Technogym SpA, Gambettola, Italy).

Subjects from both groups received a structured individualized counseling (28), aimed at achieving the currently recommended amount of PA by encouraging any type of commuting, occupational, home and leisure-time (LT) PA. Counseling was reinforced at each intermediate visit (i.e. every 3 months) in individual sessions.

Both groups received standard medical care consisting of a treatment regimen aimed at achieving optimal glycemic, lipid, blood pressure (BP) and body weight targets, as established by current IDF guidelines (29) and including glucose-, lipid- and BP-lowering agents as needed. For ethical reasons, drugs were also adjusted throughout the study to attain target levels and to account for reduced needs. Since all patients were overweight or obese, caloric intake (55% complex carbohydrates, 30% fat, and 15% protein) was reduced to obtain a negative balance of 500 kcal/day. Requirements were calculated by adding the estimated energy expenditure from PA to basal metabolism (24). Adherence to diet was verified by the use of food diaries and was similar in control and exercise group and also in LI and HI participants. Dietary prescriptions were adjusted at each intermediate visit.

### Outcomes

The primary outcome was HbA_1c_ reduction, whereas secondary outcomes included: other modifiable CVD risk factors and total and fatal coronary heart disease (CHD) 10-year risk scores; dosage of glucose-, lipid- and BP-lowering drugs; volume of PA, both non-supervised and, when applicable, supervised; physical fitness; and physical and mental health-related quality of life.

The main analyses comparing these endpoints in the EXE versus CON subjects have been reported previously (9,30–32). Here, we present a pre-specified sub-analysis comparing HI versus LI participants in the EXE group with respect to changes in HbA_1c_, other modifiable CVD risk factors and CHD 10-year risk scores.

#### Cardiovascular risk factors

The following modifiable CVD risk factors were evaluated at baseline and end-of-study: HbA_1c_, fasting blood glucose and serum insulin, Homeostasis Model Assessment-Insulin Resistance (HOMA-IR) index, waist circumference, body mass index (BMI), BP, triglycerides, total, HDL and LDL cholesterol, and high sensitivity-C-reactive protein. Biochemical tests were performed at the central laboratory, at baseline and end-of-study, and locally, throughout the study period, in order to adjust treatment regimen (9,24). HOMA-IR index was calculated using the HOMA Calculator available at http://www.dtu.ox.ac.uk/homacalculator/index.php, whereas LDL cholesterol was estimated by the equation: LDL cholesterol (in mmol/L) = total cholesterol – [HDL cholesterol+(triglycerides/2.17)], as previously reported (24). Total and fatal CHD 10-year risk scores were calculated using the United Kingdom Prospective Diabetes Study risk engine (33).

#### Volume of PA

At baseline, the volume of PA was assessed retrospectively using the Minnesota LTPA questionnaire (34). The amount of non-supervised PA was prospectively evaluated throughout the study by asking patients to fill in a daily diary, which was preliminary validated by test-retest reliability. This diary considered the list of PAs coded in the Minnesota questionnaire. Volume was calculated by multiplying the metabolic equivalent (MET, corresponding to a metabolic rate of 1 kcal or 3.5 ml O_2_/kg body weight/min) scores of each Minnesota code (35) by time spent in each activity and expressed as METs-hr·wk^-1^. For supervised aerobic exercise, energy expenditure during supervised sessions was calculated automatically by the machines from workload (i.e. the combination of speed and slope for treadmill, steps per minute for step and power for ergometer), using the ACSM’s equations (36). For supervised resistance exercise, an estimate of 3 METs-hr was established, based on direct measurements in patients with type 2 diabetes showing this amount of caloric expenditure, independent of training intensity (37).

#### Physical fitness

Parameters of physical fitness, i.e. cardio-respiratory fitness, strength and flexibility, were evaluated at baseline, end-of-study, and, in the EXE group, also during the study period, in order to adjust training loads (24,32). Assessment of cardio-respiratory fitness consisted of a sub-maximal VO_2max_ evaluation, i.e. at 80% of the predicted maximal heart rate. All patients performed the test at the treadmill, using a modified Balke and Ware protocol (38), with direct measurement of oxygen consumption using the gas exchange analyzer FitMate (Cosmed, Rome, Italy) and concurrent assessment of heart rate. For strength assessment, though the 1-RM is the most reliable test, we used a maximal repetition (or 5–8 RM) test, which is preferable in patients with a low fitness profile for safety reasons, and then predicted 1-RM using the Brzycki formula (39). For hip and trunk flexibility assessment, a standard bending test was performed.

#### Adverse events

Adverse events were reported at supervised sessions or intermediate visits, by completing a standard form.

### Statistical Analysis

A post-hoc power calculation showed that the actual sample size provided an 84% power to detect a between-group mean difference of 0.5% in HbA_1c_ levels (α = 0.05), given an observed baseline SD of HbA_1c_ of 1.4%.

Data were expressed as mean (SD) or number of cases (percentage). Mean differences (95% confidence intervals) between baseline to end-of-study changes in the two subgroups were also reported. The efficacy of HI versus LI training on primary and secondary endpoints was assessed using the unpaired t-test or the Mann Whitney U test for continuous variables, by comparing between-groups changes from baseline to end-of-study. For categorical variables (i.e. medications), logistic regression analysis was applied, with end-of-study rate of use included in the model as the dependent variable and baseline rate of use and study arm included as covariates.

Within-group end-of-study versus baseline values were compared using the McNemar test for categorical variables and the Wilcoxon signed ranks test for continuous variables. To assess whether intensity was an independent predictor of changes from baseline in a given parameter, a multiple regression analysis was applied, with baseline values of that parameter, duration of diabetes, and volume of PA/exercise as covariates forced in the model. Results were expressed as beta unit coefficients.

To account for change in medication throughout the 12-month period, we performed multiple regression analysis, with baseline to end-of-study changes in a given CVD risk factor as dependent variable. Treatment at baseline and treatment initiation during the study were included in the model as dichotomous variables (yes versus no), whereas drug dosage was not taken into consideration.

The likelihood to achieve IDF targets (29) after 12 months according to subgroup, independent of volume, was estimated using a separate logistic regression model for each target (dependent variable), with study arm and baseline status (on-target versus not-on-target) as covariates. Results were expressed as odds ratios with their 95% confidence intervals.

All analyses were performed on individuals completing the follow-up; an analysis with baseline values carried forward was also applied. Statistical analyses were performed using SPSS version 13.0 (SPSS Inc., Chicago, Illinois, USA).

## Results

The median exercise training attendance was 83.3% (interquartile range, 62.8% to 85%) in the LI group and 81.3% (61.5% to 88%) in the HI group. During the 12-month period, 15 EXE subjects dropped out, 6 in the LI and 9 in the HI subgroup (Figure 1). The results presented here refer to patients completing the follow-up (LI = 136; HI = 152), since baseline values carried forward analysis did not change the estimates. Baseline characteristics of study subjects are reported in Table 1 and Supplemental Table 1 (see Supplemental Table S4). Briefly, patients in the LI and HI subgroups had 58.4 (8.9) and 59.5 (8.3) years of age, 5.9 (4.0) and 7.8 (6.2) years of diabetes duration, and a male/female ratio of 86/50 and 88/64, respectively.

**Table 1:** Physical activity, physical fitness, anthropometric, clinical and biochemical parameters and risk scores at baseline and at the end of the 12-month study period.

	LI	LI	*P* value*	HI	HI	*P* value*	Mean difference	*P* value †
	Baseline	12 months	0–12 months	Baseline	12 months	0–12 months	(95% CI)	HI vs. LI
**PA data**								
**Non-supervised PA,** **METs-hr. wk^-1^**	0.67 (1.78)	12.49 (7.22)	<0.001	0.79 (1.90)	12.51 (7.67)	<0.001	−0.02 (1.76, −1.71)	1.000
**Supervised PA, METs-hr. wk^-1^**	0.00 (0.00)	7.80 (3.27)	<0.001	0.00 (0.00)	7.35 (3.63)	<0.001	−0.45 (−1.26,0.35)	0.580
**Total PA ‡, METs-hr. wk^-1^**	0.67 (1.78)	20.29 (8.50)	<0.001	0.79 (1.90)	19.86 (9.50)	<0.001	−0.82 (−2.90,1.26)	0.460
**Physical fitness data**								
**VO_2max_, ml/Kg/min**	25.1 (5.4)	29.6 (5.6)	<0.001	26.5 (5.3)	31.1 (5.9)	<0.001	0.14 (−0.65,0.92)	0.866
**Upper body strength, Kg**	41.8 (16.6)	50.3 (19.0)	<0.001	38.7 (16.0)	51.6 (19.0)	<0.001	4.37 (2.04,6.70)	0.001
**Lower body strength, Kg**	115.2 (68.3)	142.7 (75.3)	<0.001	101.6 (60.5)	137.2 (70.7)	<0.001	8.13 (−0.56,15.7)	0.023
**Bending, cm**	13.2 (10.3)	7.6 (9.7)	<0.001	12.0 (9.6)	5.9 (9.1)	<0.001	−0.49 (−1.96,0.98)	0.116
**Anthropometric and clinical data**								
**Waist circumference, cm**	107.3 (12.0)	103.5 (11.6)	<0.001	103.4 (11.2)	99.4 (10.8)	<0.001	−0.21 (−1.19,0.77)	0.981
**BMI, Kg/m^2^**	31.9 (4.7)	30.9 (4.6)	<0.001	31.2 (4.6)	29.7 (4.2)	<0.001	0.18 (−0.21,0.56)	0.169
**Systolic BP, mmHg**	140.2 (19.5)	133.2 (14.5)	<0.001	139.9 (16.6)	131.8 (13.7)	<0.001	−1.18 (−4.80,2.44)	0.534
**Diastolic BP, mmHg**	84.6 (11.7)	81.3 (8.5)	<0.001	82.6 (8.6)	79.6 (7.4)	<0.001	0.33 (−1.81,2.46)	0.886
**Biochemical data**								
**HbA_1c_, %**	6.99 (1.39)	6.66 (1.19)	0.005	7.24 (1.39)	6.74 (1.04)	<0.001	−0.17 (−0.44,0.10)	0.029
**Fasting blood glucose, mmol/l**	7.53 (2.40)	7.16 (2.14)	0.030	8.31 (2.74)	7.30 (2.26)	0.003	−0.48 (−1.14,0.18)	0.475
**Serum insulin, pmol/l**	94.5 (52.8)	82.0 (45.8)	0.009	79.2 (59.0)	74.3 (56.3)	0.03	7.2 (3.6,18.0)	0.388
**HOMA-IR**	4.62 (3.32)	3.88 (2.74)	0.009	4.40 (3.83)	3.70 (3.08)	0.003	0.69 (−0.71,0.85)	0.992
**Triglycerides, mmol/l**	1.43 (1.10)	1.51 (0.84)	0.010	1.52 (1.09)	1.48 (1.00)	0.510	0.12 (−0.34,0.10)	0.018
**Total cholesterol, mmol/l**	5.04 (0.83)	4.70 (0.76)	<0.001	5.27 (0.81)	4.68 (1.02)	<0.001	−0.24 (−0.46,-0.01)	0.037
**HDL cholesterol, mmol/l**	1.10 (0.27)	1.20 (0.27)	<0.001	1.22 (0.31)	1.30 (0.33)	<0.001	−0.04 (−0.10,0.03)	0.237
**LDL cholesterol, mmol/l**	3.31 (0.84)	2.80 (0.64)	<0.001	3.39 (0.78)	2.71 (0.84)	<0.001	−0.16 (−0.37,0.04)	0.100
**hs-CRP, mg/l**	2.95 (2.14)	2.11 (1.87)	<0.001	2.63 (2.34)	1.94 (1.97)	<0.001	0.15 (−0.32,0.61)	0.095
**10-yr UKPDS risk scores**								
**Total CHD**	19.3 (13.6)	15.6 (9.9)	<0.001	19.7 (13.3)	15.9 (10.9)	<0.001	−0.17 (−1.84,1.50)	0.645
**Fatal CHD**	12.2 (11.1)	9.7 (7.9)	<0.001	13.0 (10.8)	10.4 (8.7)	<0.001	−0.22 (−1.56,1.12)	0.532

Values are mean (SD); LI = low-intensity subgroup; HI = high-intensity subgroup; PA = physical activity; METs = metabolic equivalents; VO_2max_ = maximal oxygen consumption; BMI = body mass index; BP = blood pressure; HOMA-IR = Homeostasis Model Assessment-Insulin Resistance; hs-CRP = high sensitivity C-reactive protein; UKPDS = United Kingdom Prospective Diabetes Study; CHD = coronary heart disease.

* Wilcoxon signed ranks test; † Mann Whitney U-test; ‡ Total PA = non-supervised+supervised PA.

**Figure 2:**
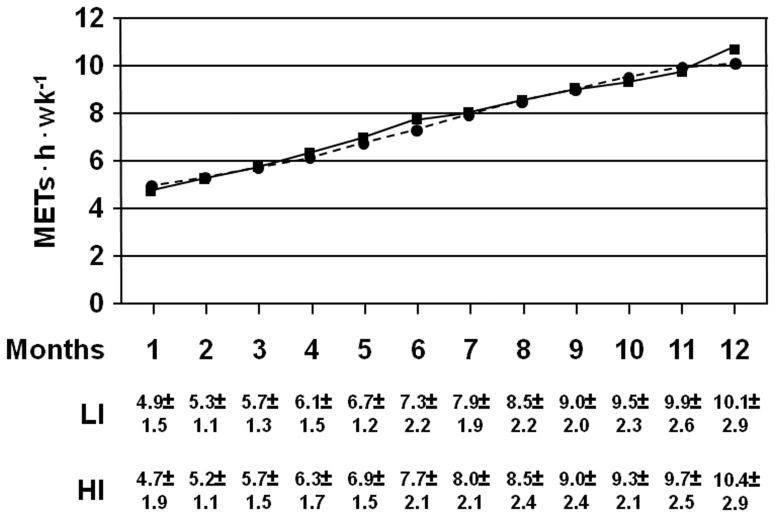
Average monthly energy expenditure from supervised exercise. Energy expenditure data in LI (circles, dotted line; n = 136) and HI (squares, continuous line; n = 152) participants [mean values; means (SD) are reported below]. LI = low intensity; HI = high intensity.

According to the study protocol, duration of aerobic training increased from 46 min at month 1 to 53 min at month 12 in the LI subgroup and from 34 min at month 1 to 40 min at month 12 in the HI subgroup. Conversely, duration of resistance training was identical in the two subgroups and did not change throughout the study (30 min), since pauses were longer in HI than in LI subjects, and lift values, but not number of series or repetitions, varied with improvements of predicted 1-RM, As a consequence, duration of each session increased from 76 min at month 1 to 83 min at month 12 in the LI subgroup and from 64 min at month 1 to 70 min at month 12 in the HI subgroup. Moreover, energy expenditure during supervised sessions was similar in the two subgroups (Figure 2). Average volumes during the 12-month period were 7.58±1.92 and 7.60±2.02 in LI and HI participants, respectively. Also the volume of unsupervised PA did not differ between LI and HI subjects. Conversely, changes over baseline in upper and lower body strength, but not in VO_2max_ and flexibility, were significantly more marked in the HI versus LI subgroup, though all fitness parameters improved in both subgroups (Table 1).

Reduction in the primary endpoint HbA_1c_ was slightly, though significantly higher in HI than in LI subjects as a result of a 0.50% decrease in the former versus a non-significant 0.33% reduction in the latter subgroup. In addition, both LI and HI subjects exhibited significant improvements in all the other CVD risk factors except triglycerides, which increased in LI and decreased non-significantly in HI participants, and also in CHD risk scores. However, changes over baseline were significantly more marked in HI than in LI subgroup for triglycerides and total cholesterol, but not for fasting blood glucose, insulin, HOMA-IR index, systolic and diastolic BP, waist, BMI, HDL and LDL cholesterol, high sensitivity-C-reactive protein, and total and fatal CHD risk scores (Table 1). Indeed, at multiple regression analysis, intensity was not an independent predictor of reduction of either HbA_1c_ (β = −0.05; P = 0.64), triglycerides (β = −6.28; P = 0.43) or total cholesterol (β = –4.28; P = 0.27). Results did not change when duration of diabetes, which was different in the two subgroups, was included in the model as covariate (not shown).

**Table 2:** Medications at baseline and at the end of the 12-month study period.

	LI	LI	*P* value*	HI	HI	*P* value*	*P* value †
	Baseline	12 months	0–12 months	Baseline	12 months	0–12 months	HI vs. LI
**Diet alone**	8 (5.9)	8 (5.9)	1.000	17 (11.2)	13 (8.6)	0.220	0.310
**Oral hypoglycemic agents**	113 (83.1)	114 (83.8)	1.000	123 (80.9)	126 (82.9)	0.450	0.740
**Sulfonylureas**	27 (19.9)	27 (19.9)	1.000	50 (32.9)	45 (29.6)	0.270	0.770
**Glinides**	19 (14.0)	20 (14.7)	1.000	10 (6.6)	11 (7.2)	1.000	0.670
**Metformin**	100 (73.5)	99 (72.8)	1.000	113 (74.3)	117 (77.0)	0.220	0.190
**Thiazolidinediones**	10 (7.4)	20 (14.7)	0.006	18 (11.8)	27 (17.8)	0.010	0.710
**Acarbose**	2 (1.5)	2 (1.5)	1.000	3 (2.0)	3 (2.0)	1.000	1.000
**Insulin**	19 (14.0)	20 (14.7)	1.000	18 (11.8)	23 (15.1)	0.270	0.320
**Alone**	8 (5.9)	9 (6.6)	1.000	10 (6.6)	9 (5.9)	1.000	0.610
**Combined**	11 (8.1)	11 (8.1)	1.000	8 (5.3)	14 (9.2)	0.110	0.150
**Anti-hypertensive agents**	89 (65.4)	86 (63.2)	0.380	105 (69.1)	105 (69.1)	1.000	0.250
**ACE inhibitors**	46 (33.8)	45 (33.1)	1.000	52 (34.2)	48 (31.6)	0.290	0.300
**Angiotensin II-receptor antagonists**	35 (25.7)	34 (25.0)	1.000	40 (26.3)	41 (27.0)	1.000	0.340
**Diuretics**	35 (25.7)	31 (22.8)	0.220	38 (25.0)	36 (23.7)	0.630	0.740
**Calcium-channel blockers**	24 (17.6)	25 (18.4)	1.000	27 (17.8)	30 (19.7)	0.380	0.710
**β-blockers**	20 (14–7)	24 (17.6)	0.130	30 (19.7)	32 (21.1)	0.690	0.060
**α_1_-blockers**	8 (5.9)	8 (5.9)	1.000	5 (3.3)	5 (3.3)	1.000	1.000
**Lipid-lowering agents**	48 (35.3)	54 (39.7)	0.070	68 (44.7)	76 (50.0)	0.040	0.710
**Statins**	37 (27.2)	43 (31.6)	0.070	54 (35.5)	63 (41.4)	0.004	0.330
**Fibrates**	7 (5.1)	8 (5.9)	1.000	10 (6.6)	10 (6.6)	1.000	0.630
**ω-3**	8 (5.9)	10 (7.4)	0.630	10 (6.6)	12 (7.9)	0.730	0.400

Values are n (%); LI = low-intensity group; HI = high-intensity group.

* McNemar test; † Logistic regression adjusted for baseline.

When compared with the CON group, changes over baseline in both the LI and HI subgroups were significantly more marked for HbA_1c,_ HOMA-IR, serum insulin, systolic and diastolic BP, total, HDL- and LDL-cholesterol, waist, BMI, high sensitivity-C-reactive protein, and total and fatal CHD 10-year risk scores (not shown), as previously reported for comparison of the whole EXE group with CON participants (9).

**Table 3:** Subjects on-target for traditional cardiovascular risk factors at baseline and at the end of the 12-month study period and probability of reaching targets at 12 months.

Target	LI	LI	*P* value*	HI	HI	*P* value*	OR (95% CI) †
	Baseline	12 months	0–12 months	Baseline	12 months	0–12 months	HI vs. LI
**HbA_1c_<6.5%**	59 (43.4)	70 (51.5)	0.080	54 (35.5)	73 (48.0)	0.003	1.04 (0.61,1.80)
**TG<1.69 mmol/l**	107 (78.7)	99 (72.8)	0.230	113 (74.3)	111 (73.0)	0.880	1.10 (0.63,1.91)
**Total C<4.53 mmol/l**	42 (30.9)	47 (34.6)	0.530	31 (20.4)	69 (45.4)	<0.001	1.99 (1.19,3.35)
**HDL C>1.04 mmol/l**	64 (47.1)	98 (72.1)	<0.001	106 (69.7)	121 (79.6)	0.010	1.00 (0.54,1.81)
**LDL C<2.59 mmol/l**	25 (18.4)	45 (33.6)	<0.001	22 (14.4)	73 (48.7)	<0.001	2.27 (1–34,3.85)
**SBP<130 mmHg**	34 (25.0)	48 (35.3)	0.020	30 (19.7)	59 (39.1)	<0.001	1.45 (0.84,2.49)
**DBP<80 mmHg**	(28 (20.6)	35 (25.7)	0.210	24 (15.8)	41 (27.2)	0.003	1.31 (0.71,2.39)

Values are n (%); LI = low-intensity group; HI = high-intensity group; OR = odd ratio; CI = confidence interval; TG = triglycerides; C = cholesterol; SBP = systolic blood pressure; DBP = diastolic blood pressure.

* Mc Nemar test; † Logistic regression adjusted for baseline status (on-target vs. not-on-target), treatment at baseline and change during the study.

During the study period, changes in medication were similar in the HI and LI subgroups (Table 2) and adjusted multiple linear regression analysis showed that these changes did not affect differences between subgroups in variations of HbA_1c_, triglycerides and total cholesterol (not shown).

**Table 4:** Adverse events.

Events	LI	HI	*P* value
**A. Related to exercise intervention**	**15**	**19**	0.470 *
shoulder pain/chronic tendinopathy of rotator cuff	4	5	
(aggravation of) low back pain	2	4	
aggravation of pre-existing osteoarthritis of hip orknee joint	2	3	
shin splints/lower limb pain	3	4	
other/generalized musculoskeletal discomfort	4	3	
**B. Unrelated to exercise intervention**	**12**	**13**	0.780*
**1. Elective surgery:**	**7**	**6**	0.700*
arthroscopic knee surgery	1	1	
total thyroidectomy	0	0	
cataract surgery	1	1	
knee/hip joint replacement	1	1	
inguinal hernia	1	0	
varicose vein surgery	1	1	
percutaneous coronary revascularization	1	1	
mastectomy for carcinoma of the mammary gland	1	0	
percutaneous lower limb revascularization	0	1	
**2. Other serious medical event:**	**4**	**7**	0.400*
atrial fibrillation	0	1	
newly diagnosed myocardial ischaemia	1	2	
accidental bone fracture	2	1	
bronchitis/pneumonia	0	2	
Otitis	1	1	
**3. Death from any cause**	**1**	**0**	0.160 *
**4. Death from cardiovascular causes**	**0**	**0**	1.000 †
**Total**	**27**	**32**	0.460 *

* χ^2^ test; † Fisher’s exact test.

At end-of-study, the percentage of subjects on-target according to the IDF Guidelines increased significantly for HDL and LDL cholesterol and systolic BP in the LI subgroup and also for HbA_1c_, total cholesterol and diastolic BP in the HI subgroup. Overall, the probability of reaching targets was significantly higher in HI versus LI subjects only for total and LDL cholesterol (Table 3).

To further investigate the differences between the two subgroups in HbA_1c_ change from baseline and odd ratios for LDL cholesterol target, subjects with baseline HbA_1c_ >7.5% or LDL cholesterol>3.0 mmol/l were analyzed separately. Results showed that the mean differences between LI and HI participants were even lower in these patients and were in favor of subjects training at LI (0.4% [−0.60,0.69], P = 0.89, and 0.40 [−0.26,0.34] mmol/l, P = 0.78, respectively).

Adverse events related to PA were not more frequent in HI than in LI subjects, and also the number of unrelated events did not differ between the two subgroups (Table 4). No episode of hypoglycemia requiring assistance was recorded in subjects from both subgroups.

## Discussion

Exercise is being increasingly recognized as a form of therapy in subjects with type 2 diabetes, since it is effective in improving fitness and reducing HbA_1c_ and other CVD risk factors as well as the dosage/number of glucose-, lipid- and BP-lowering drugs (5–13). However, there is still debate regarding the type, volume and intensity of exercise to be prescribed to these high-risk individuals in order to reduce CVD burden. The recent ACSM/ADA position statement recommends combined aerobic and resistance, preferably supervised, training and a minimum weekly amount of 150 minutes of moderate-to-vigorous aerobic training plus 2–3 sessions of moderate-to-vigorous resistance exercise (14). While recommendations on type and volume of exercise are based on solid evidence, there is substantial lack of information about the need to train at moderate-to-vigorous intensity for subjects with type 2 diabetes, who are difficult to engage in such programs because of their sedentary habits and frequent suffering from muscle weakness, reduced exercise tolerance, and co-morbidities.

This multicenter trial, which is of larger size and longer duration than other exercise intervention trials in patients with type 2 diabetes (6–8,11–13), is the first study of adequate statistical power addressing the issue of the effect of different exercise intensities at the same energy cost in these individuals. It showed that training at HI does not provide significant incremental benefits, as compared with iso-volumetric LI training, in term of improvements of modifiable CVD risk factors and estimated CHD risk, with the exception of HbA_1c_, triglycerides and total cholesterol. Moreover, differences between the two subgroups in these parameters, though statistically significant, were marginal (0.17% for HbA_1c_, 0.12 mmol/l for triglycerides, and 0.24 mmol/l for total cholesterol) and hence not clinically relevant. This interpretation is supported by the findings that intensity was not an independent predictor of reduction of either HbA_1c_, triglycerides or total cholesterol and that the probability of reaching targets for these variables, except cholesterol, was similar in LI and HI subjects. However, since outcome measures were obtained only at baseline and end-of-study, it could not be determined whether the rate of improvement was different between the two subgroups.

These data are in keeping with a previous small-sized study in subjects with type 2 diabetes participating in a continuous endurance-type exercise program (16) and also with a larger trial in overweight women engaged in an unsupervised aerobic training program (21). Moreover, our results support the concept that type and volume of exercise (9,11–13) are more important than intensity in targeting glycemic control and reduction of CVD risk in these individuals. On the other hand, the very low rate of dropouts and adverse events in the two subgroups indicates that both modalities are safe in these individuals who usually suffer from several co-morbidities. Thus, since intensity was varied by changing the duration of aerobic training and the number of repetitions of resistance training in order to obtain the same work volume (or caloric expenditure), the comparable efficacy and safety of the two modalities imply that patients may choose to train at LI or HI depending on factors such as time, fatigue and personal preference.

There are two possible reasons for lack of additional benefit from more vigorous exercise. As in previous studies (16–22), only supervised exercise was performed at LI or HI, since, in case of individuals with type 2 diabetes, who are often elderly, sedentary, and suffering from various co-morbidities, working at HI in the absence of supervision is not recommended for safety reasons. Thus, due to the high volume of unsupervised PA achieved by both subgroups as a result of a successful counseling intervention, only 1/3 of total PA was performed at different intensities. More importantly, 15–20% differences in intensity in low-fitness individuals such as sedentary subjects with type 2 diabetes might not translate into absolute differences in aerobic and resistance workloads which are large enough to produce clinically significant differences in HbA_1c_ and other modifiable CVD risk factors. However, with increasing fitness and consequent adjustments of intensity based on improvements in predicted VO_2max_ and 1-RM, absolute workloads tended to diverge more markedly with time. Thus, it is possible that, with a longer duration of the study, significant differences between the two subgroups would have emerged, due to the progressively more pronounced differences in absolute workloads, not compensated by opposite differences in the duration of aerobic training and number of series of resistance training. This view is consistent with the finding that working at HI versus LI resulted in significantly more marked improvements in muscular fitness, which was shown to be inversely and independently related to all-cause mortality in the general population (40) and also to HbA_1c_ levels and other modifiable CVD risk factors in subjects with type 2 diabetes (32,41). In addition, it is possible that training at>70% of predicted VO_2max_ and at>80% of predicted 1-RM, i.e. outside the range indicated by the guidelines (10,14,27), would be more effective in reducing modifiable CVD risk factors, thus becoming the preferred training modality, provided that it is not associated with reduced safety.

Strengths of this study are the large size, the long duration, and, particularly, the ability to isolate the effects of 2 different exercise intensities at a fixed volume (or energy cost) (23). A limitation is that, though patients received specific dietary prescriptions and adherence to diet was verified at intermediate visits, diet was not considered in data analysis.

In conclusion, data from the IDES indicate that, at least in low-fitness individuals such as sedentary subjects with type 2 diabetes, training at LI is approximately as effective as training at HI in improving modifiable CVD risk factors and reducing CVD burden, thus suggesting that, for practical purposes, intensity is a less important issue than volume and type of training when exercise is applied as a form of therapy in these individuals.

## Supporting Information

The IDES Investigators S1 **List of the IDES Investigators.**
(DOC)

IDES Protocol S1 **Trial protocol.**
(DOC)

Checklist S1 **CONSORT checklist.**
(DOC)

Table S1 **Supplemental Table 1.**
(DOC)
