# Supplementary material for: Effect of High- versus Low-Intensity Supervised Aerobic and Resistance Training on Modifiable Cardiovascular Risk Factors in Type 2 Diabetes; The Italian Diabetes and Exercise Study (IDES)
Source: PLoS One. 2012 Nov 21;7(11):e49297. doi: 10.1371/journal.pone.0049297 (PMC3504024; doi:10.1371/journal.pone.0049297)
Supplement: The IDES Investigators S1 — List of the IDES Investigators. (DOC) [file pone.0049297.s001.doc]

**The IDES investigators S1**

**Diabetes Centers**

1. Diabetes Division, S. Andrea Hospital, Rome: Francesco Fallucca MD; Giuseppe Pugliese MD, PhD; Maria Cristina Ribaudo MD; Elena Alessi, MD; Paola Simonelli, MD; Laura Salvi, MD, Alessandra Bazuro, MD; Luca Pugliese, BA; Carla Maccora, BS.
2. Division of Endocrinology and Metabolism, National Geriatric Institute (I.N.R.C.A.), Rome, Felice Strollo MD; Massimo Morè MD
3. Diabetes Unit, Villa S Pietro Hospital: Pietro Alimonti MD; Nicolina Di Biase MD; Filomena Lasaracina MD
4. Diabetes Unit, City Hospital of Civitavecchia: Graziano Santantonio MD
5. Diabetes Prevention Center (ACISMOM) Latina: Laura Cruciani MD
6. Diabetes Unit, “Triolo Zancla” Clinic, Palermo: Mario Manunta MD
7. Research Center for Physical Activity in Diabetes, University of Catania: Maurizio Di Mauro MD
8. Department of Experimental and Clinical Medicine. University of Catanzaro: Giorgio Sesti MD, Concetta Irace MD; and Diabetes Unit, City Hospital of Catanzaro: Luigi Puccio MD
9. Division of Endocrinology and Metabolism, University of Foggia: Mauro Cignarelli MD; Vincenzo Nicastro MD; Sabrina Piemontese MD
10. Diabetes Unit (AID), Provincial Health Authority for Naples1: Gerardo Corigliano MD; Ernesto Rossi MD; Marco Corigliano MD
11. Department of Internal Medicine,University of Perugia: Pierpaolo De Feo MD;Cristina Fatone MD
12. Diabetes Unit, New Hospital of San Giovanni di Dio, Florence: Cristiana Baggiore MD; Roberto Russo MD
13. Division of Diabetes and Metabolism, National Geriatric Institute (I.N.R.C.A), Ancona: Massimo Boemi MD; Luigi Lanari MD
14. Diabetes Unit, City Hospital of Brescia: Umberto Valentini MD; Angela Girelli MD
15. Diabetes Unit, City Hospital of Ravenna: Paolo Di Bartolo MD; Francesca Pellicano MD
16. Diabetes Unit, City Hospital of Rimini: Paolo Mazzuca MD
17. Diabetes Unit, City Hospital of Reggio Emilia: Enrica Manicardi MD
18. Diabetes Unit, San G. Battista Hospital, Turin: Alberto Bruno MD
19. Diabetes Unit, “Cà Foncello” Hospital, Treviso: Maria Sambataro MD
20. Diabetes Clinic, Monterotondo, Rome: Stefano Balducci MD
21. Department of Internal Medicine, San Paolo Hospital, University of Milan: Antonio Pontiroli MD; Marco Laneri MD; Anna Boggio MD
22. Diabetes Unit, Belcolle Hospital, Viterbo: Nunzio Zagari MD

**Metabolic Fitness Centers**

1. Center for Functional Assessment in Sport, Sant’Andrea Hospital, Rome: Carla Iacobini, Stefano Menini
2. Health Care Team, Monterotondo: Gianluca Balducci, Lorella Senigagliesi, Enza Spinelli
3. Athlos Club, Civitavecchia: Alessandro Di Giovanni
4. Metabolic Fitness, Latina: Mariano Pineda
5. DO-IN, Palermo: Umberto Pandolfo
6. The Wellness Center, Naples: Cristina De Fazio
7. Elisir Club, Perugia: Antonella Settequattrini
8. Metagym, Florence: Marco Gambacciani
9. Fisioclub, Ancona: Matteo Fabrizi
10. Lifeplanet, Ravenna: Adriano Ceccherini, Enrico Balducci
11. Steven Sporting Club, Rimini: Mirko Quattrini
12. Cemter for Physiatrics and Sports Medicine VITALIA, Torino: Massimo Massarini
13. Pianeta sport, Treviso: Giuseppe Baggio
14. Winner Clubs, Reggio Emilia: Marco Fornari
15. Millennium, Brescia: Davide Violi
16. GCube Fitness & Wellness, Viterbo: Giancarlo Cherubini
17. Zenith Center for Medicine, Foggia: Fabio Mastelloni
18. S.S. Free Studios UISP, Catanzaro: Valeria Micali
19. Research Center for Physical Activity in Diabetes, Catania: Carmelo D’Urso
20. Centro “La fonte del benessere”, Milan: Davide Canevari
