# Supplementary material for: Effect of High- versus Low-Intensity Supervised Aerobic and Resistance Training on Modifiable Cardiovascular Risk Factors in Type 2 Diabetes; The Italian Diabetes and Exercise Study (IDES)
Source: PLoS One. 2012 Nov 21;7(11):e49297. doi: 10.1371/journal.pone.0049297 (PMC3504024; doi:10.1371/journal.pone.0049297)
Supplement: IDES Protocol S1 — Trial protocol. (DOC) [file pone.0049297.s002.doc]

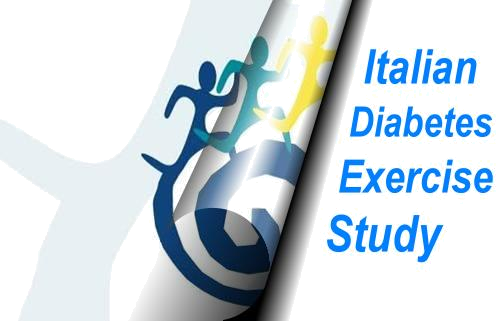


**RESEARCH PROTOCOL S2**


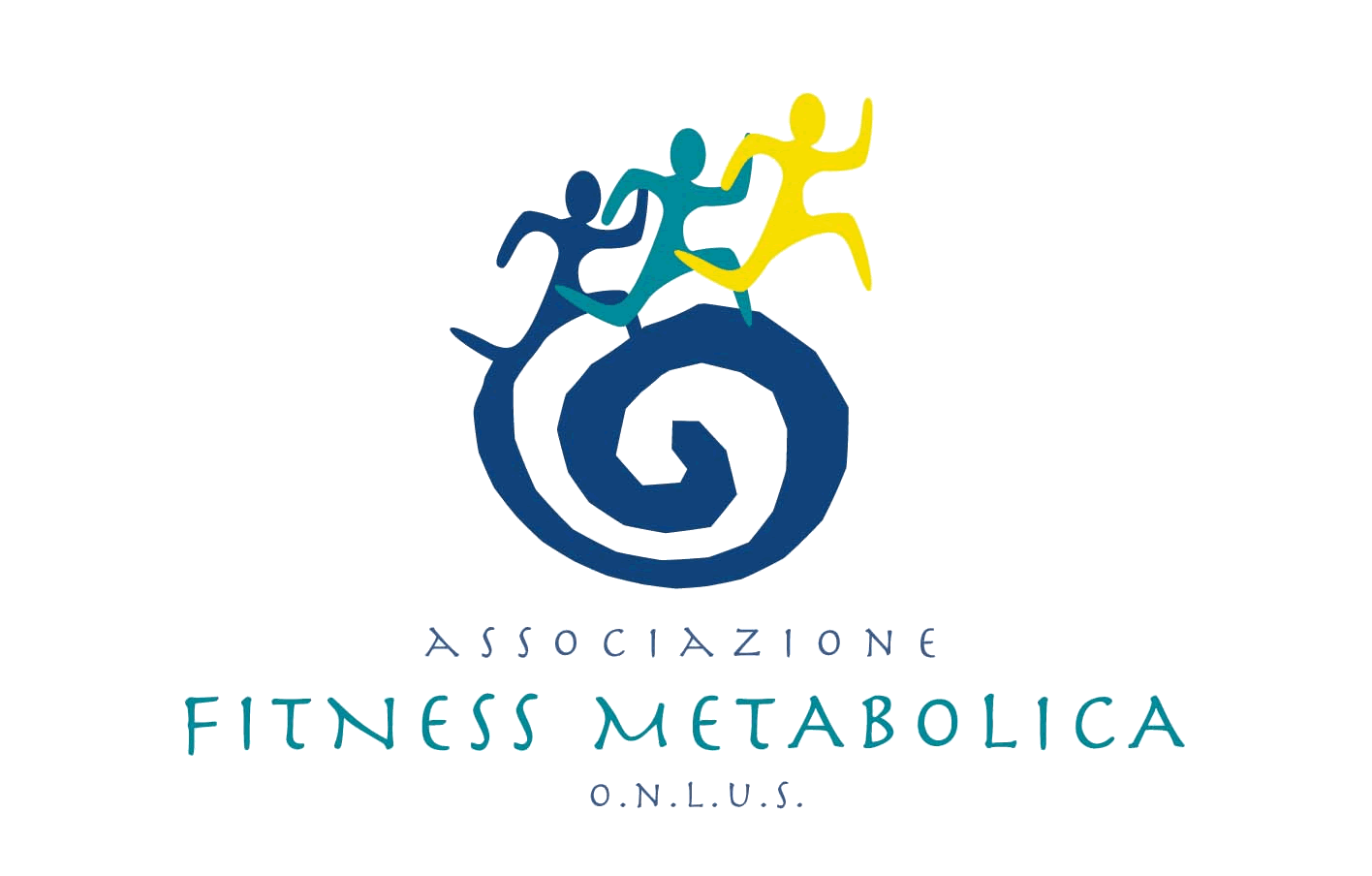

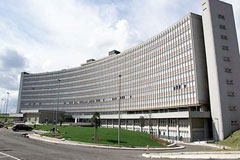

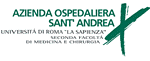

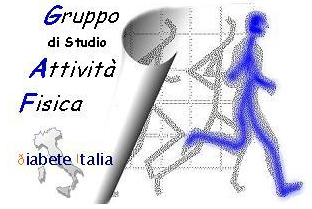


**Background**

Prevalence of diabetes mellitus, particularly type 2, is rapidly increasing worldwide and, though the rise is more pronounced in developing countries, Europe and North America are not spared by this “epidemics”. It has been estimated that there will be about 221 millions diabetic subjects in the world in 2010, as compared with the 124 millions patients estimated in 1997 [1]. As a consequence, also the burden of the invalidating long-term complications of diabetes is expected to rise, with an inevitable increase of the social and economic costs associated with the disease.

Diabetic subjects are in fact at higher risk for macro and microvascular disease than the general population [2]. The increased morbidity and mortality for cardiovascular disease (CVD) has been attributed to both chronic hyperglycemia *per se* and comorbid conditions occurring in diabetic patients more frequently than in nondiabetic subjects. These conditions include central obesity, dyslipidemia and arterial hypertension, which have been shown to cluster and associate with insulin resistance and disturbances of glucose metabolism, thus suggesting the existence of a unique pathophysiological condition, called “Metabolic Syndrome” (MS) or “insulin resistance syndrome” [3].

Sedentary habits have been associated with an increased prevalence of diabetes and MS. Moreover, a low cardiorespiratory fitness was shown to be a powerful and independent predictor of all-cause and CVD mortality in type 2 diabetic subjects [4-7]. Conversely, lifestyle-modification programs including physical activity were shown to prevent the development of type 2 diabetes in high risk subjects; this was associated with significant improvements in body weight, central adiposity, HbA1c and lipid levels, insulin sensitivity and prevalence of hypertension [8-13]. Moreover, in type 2 diabetic patients, a moderate or high level of physical activity (occupational, commuting, and leisure-time) reduced total and CVD mortality by improving glycemic control and other CVD risk factors [14,15].

The Italian Diabetes and Exercise Study (IDES) has been designed to evaluate the efficacy on modifiable CVD risk factors of a prescribed and supervised mixed (aerobic and resistance) training program, in addition to structured exercise counseling as part of conventional disease management, as compared with standard care alone (including structured exercise counseling), in type 2 diabetic subjects with the metabolic syndrome (MS).

# **Design and methods**

# **Study design**

# This study is a randomized controlled trial (ISRCTN-04252749). The protocol complies with the Declaration of Helsinki.

**Endpoints**

***Primary outcome***

The primary research goal is the evaluation of the efficacy of a mixed exercise training program, prescribed and supervised, as compared with standard care, in terms of reduction of HbA_1c_ levels.

***Secondary outcomes***

Secondary research goals include an exploration of :

- Percentage of patients in each group that after 12 month achieve one or more of the following targets: HbA_1c_ <6.5% or HbA1c reduction >0.5%; total cholesterol <175 mg/dl; LDL cholesterol <100 mg/dl; HDL cholesterol >40 mg/dl; triglycerides <150 mg/dl; systolic blood pressure (BP) <130 mmHg; diastolic BP <80 mmHg; BMI reduction >1; waist circumference reduction >5 cm.
- Difference between groups in terms of reduction of HbA_1c_, serum triglyceride and LDL-cholesterol, systolic and diastolic BP, BMI and waist circumference and increment of serum HDL-cholesterol.
- Difference between groups in terms of reduction of number and/or dosage of glucose-, lipid- and BP-lowering drugs.
- Difference between groups in terms of reduction of global cardiovascular 10-year risk (UKPDS risk engine tables).
- Difference between groups in terms of improvement of well-being.
- Difference between groups in terms of direct medical costs and direct and indirect social costs.
- Dose/response relationship between the quality (volume and intensity) of exercise training and modifiable cardiovascular risk factors.
- Relationship between physical fitness at baseline and during the study and modifiable cardiovascular risk factors.

# ***Eligibility criteria***

# The inclusion and exclusion criteria are summarized in Table 1. The main entry criterion is known type 2 diabetes plus >2 other MS traits, as defined according to the IDF criteria [16], i.e. waist circumference M >94 cm F >80 cm; triglycerides >150 mg/dl and/or HDL cholesterol M <40 mg/dl F <50 mg/dl or specific treatment; and BP >130/85 mmHg or specific treatment. Additional requirements are age from 45 to 75 years, duration of diabetes > 1 year, BMI 27-40 kg/m^2^, sedentary lifestyle for at least six months, ability to walk without assistance and eligibility after cardiovascular evaluation. Patients having any condition that limits or contraindicates the exercise will be excluded from the study.

# ***Recruitment, randomization and baseline evaluation***

# Patients fulfilling the above criteria will be recruited in 22 outpatients clinics nationwide (see Appendix 1 and Figure 1) and subsequently randomized to two arms:

# mixed (aerobic+ resistance) exercise, prescribed and supervised + exercise counseling (EXE);

# conventional treatment, including exercise counseling (CON);

# for 12 months.

# All patients attending the outpatients clinics participating in this study will be evaluated for eligibility. The recruitment process will include 3 visits designated as R1, R2 and R3 (Table 2)

# During the first visit (R1), eligible patients will be registered in the IDES database, together with their demographic, anthropometric and clinical data, and will be asked to participate in the study; those who accept will be invited to read and sign a statement of informed consent (see Appendix 2), whereas those who do not accept will be excluded from the study. Each patient will undergo two specialist consultations: a visit of an ophthalmologist for funduscopic evaluation and a cardiologic examination for resting ECG and an ECG treadmill test for cardiovascular disease (CVD) evaluation when needed.

# On the second visit (R2), after excluding from the study those patients who will be eventually found to have severe retinopathy or CVD on the ophthalmologic and cardiologic visits, all subjects will be asked to fill in two forms, the ITALIAN SF-36 health survey (ISF-36) [17] and the WHO – Diabetes Treatment Satisfaction Questionnaire (WHO-DTSQ) [18] for baseline assessment of subjective health status perception and diabetes treatment satisfaction. A structured individualized counseling intervention [19] will be then administered to all patients, independent of the group assignment, and blood and urine samples will be collected and sent via express courier to the central laboratory for being assayed for group A biochemical measurements at baseline (Table 3). The Minnesota Leisure Time Physical Activity (LTPA) questionnaire [20] will be also given to all patients for retrospective assessment of the volume of physical activity at baseline. Finally, patients will be informed about group assignment, as established by the use of a centralized permuted-block randomization computer program and subsequently assessed at the Metabolic Fitness Center for basal evaluation of physical fitness.

# On the third visit (R3), basal anthropometric and blood pressure (BP) measurements will be obtained and registered in the database, together with results of laboratory tests performed centrally. Subsequently all patients will receive a treatment regimen aimed at achieving optimal glycemic, lipid, BP and body weight targets, as established by current guidelines. This program will eventually include glucose-, lipid- and BP-lowering agents as needed and, when indicated, anti-platelet drugs. For ethical reasons, drugs will be also adjusted throughout the study to attain target levels and to account for reduced needs. The treatment regimen will include also a dietary prescription, which will be preceded by a preliminary nutritional evaluation with assessment of BMI and the individual patient requirements and preferences. The diet will contain 55% calories from complex carbohydrates, 30% from fat, and 15% from protein. Since all patients are overweight or obese, they will receive a low calorie diet (negative balance of 500 kcal/day, not <1500 Kcal in males and 1200 Kcal in females), calculated by adding the estimated energy expenditure from non-conditioning or non-volunteer and conditioning or volunteer physical activity to basal metabolism estimated using the gas exchange analyzer FitMate (Cosmed, Rome, Italy). Baseline LTPA will be calculated by multiplying the weekly hours spent in the various activities, as reported in the Minnesota LTPA questionnaire, by the metabolic equivalent (MET) score of each activity, and expressed as METs · h^–1^ · week^–1^. Patients will be also given a diary to be filled in daily with type of physical activity performed and time spent in each of them (Figure 2 and Table 4). Finally, patients will receive a list of intermediate, group B biochemical measurements, to be performed at each center laboratory immediately before the next visit (Table 3), will be prescribed.

***Follow-up and final evaluation***

During treatment period, the patients recruited will be seen at 3-month intervals for three intermediate visits and one final visit at the end of the 12-month period (Table 2).

At visits F1, F2 and F3, intermediate anthropometric and BP measurements will be obtained and registered in the database, together with the volume of physical activity performed in the previous 3 months and results of laboratory tests performed locally. Where needed, the treatment regimen will be modified according to current guidelines and changes in therapy will be registered in the database. All patients will be also given a new counseling intervention aimed at verifying, encouraging and reinforcing their compliance to diet and physical exercise, either supervised (EXE group) or non-supervised (CON group), and, except for visit F3, a prescription for intermediate, group B biochemical measurements, to be performed at the individual center laboratory immediately before the next visit (Table 3). At visit F4, final anthropometric and BP measurements will be obtained and registered in the database, together with the volume of physical activity performed during the last 3-month period. Blood and urine samples will be also collected and sent via express courier to the central laboratory for group A biochemical measurements at the end of the study (Table 3), and results will be subsequently registered in the database. Assessment of health status perception and diabetes treatment satisfaction via the ISF-36 and the WHO-DTSQ will be repeated and data will be entered in the database. Finally, patients will be given an appointment at the Metabolic Fitness Center connected to the outpatient clinics for physical fitness assessment at the end of the study.

# **Treatments**

***Exercise program***

The training program for the EXE group consists of twice a week supervised sessions of 75 min duration of mixed (aerobic and resistance) exercise, for 12 months.

Aerobic training will be performed using treadmill, step, elliptical, arm or cycle-ergometer. Transformation of maximal oxygen consumption (VO_2_max) in exercise load will be performed by the use of a software (Wellness System, Technogym SPA, Gambettola, Italy) or a dedicated spreadsheet, which allow calculation of the individual training load for each equipment from VO_2_max using the American College of Sports Medicine (ACSM) equations [21].

Resistance training will consist of 4 resistance exercises, i.e. thrust movement on the transverse plane (chest press or equivalent), traction movement on the frontal plane (lateral pull down or equivalent), squat movement (leg press or equivalent), trunk flexion for the abdominals, and three stretching positions. Intensity will be adjusted according to improvements in predicted VO_2max_ and 1RM, as recorded throughout the study, thus resulting in further increments of working loads. In addition, caloric expenditure will be increased progressively by 0.1 kcal/kg body weight/session every month (Table 5).

Subjects in the EXE group will be allocated to either moderate-intensity aerobic (at 55% of predicted VO_2max_) and resistance (at 60% of predicted 1 Repetition Maximum, RM) or high-intensity aerobic (at 70 % of predicted VO_2max_) and resistance (at 80% of predicted 1RM) exercise, depending on the centres, which have been preliminary assigned to exercise patients at moderate- or high-intensity. Exercise and duration of aerobic training and number of series will be managed to obtain the same caloric expenditure per kg body weight, independent of the intensity level of exercise.

***Exercise counseling***

Counseling consists of 7 steps, as previously reported [19]: motivation, self-efficacy, pleasure, support, comprehension, lack of impediments, diary.

**Measurements**

***Assessment of physical fitness***

Parameters of physical fitness, i.e. cardio-respiratory fitness, strength and flexibility, will be evaluated at baseline and at the end of the study by the estimation of VO_2max_, a 5-8 maximal repetition (MR) and a standard bending, respectively. In the EXE Group, measurements will be performed also during the study period, in order to adjust the training loads according to improvements in fitness parameters.

*Cardio-respiratory fitness.* Assessment of cardio-respiratory fitness will consist of a sub-maximal VO_2_max evaluation, i.e. at 80% of the predicted maximal heart rate (MHR = 220 – age). It has been preferred to a maximal test, because (a) the latter cannot be performed without a cardiologist, according to the Italian law; (b) heart rate varies linearly with VO_2_ to the point of maximum exertion, thus allowing extrapolation of the actual VO2max value. The test will be preceded by two consecutive run-in sessions to become familiar with testing devices and protocols, based on the results of a pilot study showing that tests executed during the first visit have a very low reliability, which increases significantly with tests executed at the third visit. All patients will perform the test at the treadmill, which has been preferred to the cyclo-ergometer to avoid early muscle exhaustion in untrained subjects, using a protocol modified form the Balke and Ware procedure [22] (Table 6), with direct oxygen consumption analysis and concurrent assessment of heart rate. According to our protocol, patients will walk two minutes at the speed of 4.3 Km/hour at 0 % gradient (warm-up), wearing the mask of a gas exchange analyzer FitMate. Then, protocol will proceed automatically through the steps indicated in Table 5 and terminate when the patient reaches the 80% of the predicted MHR and the metabolic fitness instructor stops the procedure using the dedicated key function on the gas analyzer. In the case of patients taking medications that affect heart rate, such as β-adrenergic blockers, the Borg Rate of Perceived Exertion scale will be used (version 1-10). Patients will be stopped at the perceived value of 5-6 (hard) because, according to ACSM’s guidelines, this value corresponds to a heart rate of 70-89%. Test duration will range from 5 to 15 minutes. In the EXE group, this protocol will be repeated every three months with the same modalities and monthly without direct readings of oxygen consumption, in order to adjust the training load throughout the exercise period. For every step exceeding the previous evaluation, the predicted VO_2max_ will be incremented of 2 ml of oxygen and the training load will be increased accordingly.

*Strength.* For strength assessment, a RM test will be performed with the following modalities: thrust movement on the transverse plane (chest press or equivalent), traction movement on the frontal plane (lateral pull down or equivalent), and squat movement (leg press or equivalent). The 1RM test is the most reliable test for evaluating the maximal dynamic strength of a muscle or group of muscles. However, due the very low fitness profile of patients enrolled in this study, a MR test (or 5-8 RM test) has been preferred, for safety (to avoid maximal loads to the joint structures) and validity (untrained subjects are not always able to properly reach their 1RM) reasons. One RM will be then predicted from the weight loaded and the number of repetitions executed after a proper warm up using the Brzycki formula [23]. Results will be expressed as upper body (average of thrust movement and lateral pull down) and lower (leg press) body strength. In the EXE group, the MR test (with 5-8 repetitions) will be repeated every three months with the same testing machine to adjust the training load throughout the exercise period.

*Flexibility.* For hip and trunk flexibility assessment, a standard bending test will be executed. Standing on a step with legs fully extended, patients will be asked to bend the torso forward to try to touch the ground with their fingertips. The test will be performed three times and the distance between the finger and the ground will be measured by the exercise specialist at the third attempt.

***Assessment of physical activity***

Prospective assessment of the volume of non-supervised physical activity throughout the study will be accomplished by the use of a diary in which patients will report daily the type(s) of physical activity performed and the time spent in each of them (Figure 2). This diary is based on the range of physical activities considered in the Minnesota Leisure-LTPA questionnaire (Table 4). These activities will be divided in conditioning or volunteer (corresponding to LTPA) and non-conditioning or non-volunteer (including commuting, occupational and home activities) [24]. Volume will be calculated by multiplying the METscores corresponding to each Minnesota code [25], by time in hours per week spent in each activity, and expressed as METs . h^-1^ .week^-1^.

For supervised aerobic exercise, energy expenditure during supervised sessions will be calculated automatically by the machines from workload (i.e. the combination of speed and slope for treadmill, steps per minute for step and power for ergometer), using the ACSM’s equations [21] (Table 7). For supervised resistance exercise, since direct measurements in patients with low physical fitness such as T2DM subjects (S. Zanuso et al., unpublished results) did not confirm that energy expenditure varies with training intensity [25], we have decided on a conservative estimate of 3 METs/hour.

***Assessment of cardiovascular risk factors***

Clinical and laboratory data will be evaluated at baseline and at the end of the study, for assessing the effect of lifestyle intervention, and also at 3, 6 and 9 months, in order to verify the adequacy of treatment regimen. Clinical parameters include body height and weight, with calculation of BMI, waist circumference and BP. Laboratory parameters include Group A biochemical measurements, to be performed at central laboratory (Laboratory of Clinical Chemistry, Sant’Andrea Hospital, Rome, Italy) and Group B biochemical measurements, to be performed in each center laboratory (Table 3).

***Assessment of costs***

Costs will be calculated using standard unit costs of the resources used, averaged on the year 2006 costs of resources in Italy (tables produced by Italian National Institute of Statistics). Cost analysis includes direct medical costs and direct and indirect social costs and is reported in U.S. dollars per capita per year, adjusted to year 2006, as previously reported [4].

*Direct medical costs*. These costs include expenses for medications and other costs usually paid by the National Health Service, including counseling intervention, laboratory testing, hospitalization, and outpatient care.

*Social costs.* Direct social costs include the value of the time that participants spend in practicing physical activity, cost of related items, transportation to exercise places, and admission to health clubs, whereas indirect social costs include time that participants report as lost from work or usual activities as a result of counseling visits, illness, or injury.

**Statistical analysis**

***Sample size calculation***

Sample size calculation will be based on the mean HbA_1c_ values (7.6±1.6 %) detected in the SFIDA population, consisting of 11,000 type 2 diabetic patients, aged 46-69 years, a HbA_1c_ reduction of at least 0.5 % in EXE vs. CON group and a statistical power of 90% (α=0.05). To this end, 215 patients per arm need to be enrolled (430 patients total). Assuming a dropout rate of up to 25%, we will recruit 600 patients. Such a sample size also guarantees a statistical power of >90% to detect changes indicated for each of the parameters in the secondary endpoints.

***Type of randomization***

Patients will be randomized centrally by the use of a permuted-block randomization computer program, stratifying the enrolled subjects by center and, within each center, by age (<60 vs. ≥60 years) and type of diabetes treatment (diet ± oral agents vs. insulin).

***Statistical analysis plan***

The χ^2^ test for categorical variables and the Student’s t test or the corresponding nonparametric Mann-Whitney test for continuous variables will be utilized to compare patients’ characteristics at baseline. The efficacy of intervention on primary and secondary endpoints will be assessed using the unpaired t-test or the Mann Whitney U test for continuous variables, by comparing between-groups changes from baseline to end-of-study. For categorical variables (i.e. medications), logistic regression analysis will be applied, with end-of-study rate of use included in the model as the dependent variable and baseline rate of use and study arm included as covariates.

To account for change in medication throughout the 12-month period, multiple regression analysis will be performed, with baseline-to-end-of-study changes as dependent variable and treatment at baseline and treatment initiation during the study as dichotomous variables (yes versus no), whereas drug dosage will not be taken into consideration.

Within group end-of-study vs. baseline values will be compared using the McNemar test for categorical variables and the Wilcoxon signed ranks test for continuous variables. To identify independent predictors of HbA_1c_ changes from baseline, a multiple regression analysis was applied, with mean value of METs, baseline HbA_1c_ values, gender, age, and changes in HOMA-IR, BMI, waist, total, LDL-cholesterol and HDL-cholesterol, and hs-CRP as covariates forced in the model.

Multivariate analysis techniques (multiple or logistic regression) will be used to adjust for differences in the distribution of one or more parameters between the two arms, whereas the ANOVA trend test will be applied for evaluating the relationship of volume and intensity of exercise training (dose/response) and fitness variation with modifiable cardiovascular risk factors.

The likelihood to achieve glycaemic and nonglycaemic targets after 12 months will be estimated using logistic regression, adjusted for baseline status (on-target vs. not-on-target).

**Organization**

***Clinical centers.*** Each of the 22 participating clinical centers have a Principal Investigator and additional staff to carry out the protocol that may include recruitment coordinators, dieticians, physicians, nurses, data collectors, and others. Moreover, each has an internal or external local Biochemistry Laboratory where the intermediate Group B biochemical measurements are performed. Finally, each clinical unit is associated with a Metabolic Fitness center, where all patients perform fitness assessment and strength and flexibility evaluation and EXE patients also the prescribed exercise program, supervised by at least one Metabolic Fitness instructor.

***Coordinating center.*** The Coordinating Center is at the Diabetology Unit of the Sant’Andrea Hospital, the teaching hospital of the II School of Medicine & Surgery of the University of Rome “La Sapienza”. It is responsible for biostatistical design, analysis, and data storage and processing. The Unit of Clinical Pathology of the same institution serves as central laboratory for baseline and final biochemical measurements.

***Steering committee.*** The Steering Committee is the representative body of the IDES Research Group. It is composed of the Study Coordinator and experts of the various aspects involved in the research (see Appendix 3).

**References**

1. Amos AF, McCarty DJ, Zimmet P: The rising global burden of diabetes and its complications: estimates and projections to the year 2010. *Diabet Med* 1997; 14 (Suppl 5):S1-S85,
2. Stamler J, Vaccaro O, Neaton JD, Wentworth D: Diabetes, other risk factors, and 12-yr cardiovascular mortality for men screened in the Multiple Risk Factor Intervention Trial. *Diabetes Care* 1993; 16:434-444
3. Alberti KG, Zimmmet PZ: Definition, diagnosis and classification of diabetes mellitus and its complications. Part 1: diagnosis and classification of diabetes mellitus provisional report of a WHO consultation. *Diabet Med* 1998; 15:539-553
4. Kohl HW, Gordon NF, Villegas JA, Blair SN: Cardiorespiratory fitness, glycemic status, and mortality risk in men. *Diabetes Care* 1992; 15:184–192
5. Wei M, Gibbons LW, Kampert JB, Nichaman MZ, Blair SN: Low cardiorespiratory fitness and physical inactivity as predictors of mortality in men with Type 2 diabetes. *Ann Intern Med* 2000; 132:605–611
6. Myers J, Prakash M, Froelicher V, Do D, Partington S, Atwood JE: Exercise capacity and mortality among men referred for exercise testing. *N Engl J Med* 2002; 346:793–801
7. Church TS, Cheng YJ, Earnest CP, Barlow CE, Gibbons LW, Priest EL, Blair SN: Exercise capacity and body composition as predictors of mortality among men with diabetes. *Diabetes Care* 2004; 27:83-88
8. Lindström J, Louheranta A, Mannelin M, Rastas M, Salminen V, Eriksson J, Uusitupa M, Tuomilehto J, for The Finnish Diabetes Prevention Study Group: The Finnish Diabetes Prevention Study (DPS). Lifestyle intervention and 3-year results on diet and physical activity. *Diabetes Care* 2003; 26:3230–3236
9. Uusitupa M, Lindi V, Louheranta A, Salopuro T, Lindström J, Tuomilehto J, for the Finnish Diabetes Prevention Study Group: Long-term improvement in insulin sensitivity by changing lifestyles of people with impaired glucose tolerance. 4-year results from the Finnish Diabetes Prevention Study. *Diabete*s 2003; 52:2532–2538
10. Carr DB, Utzschneider KM, Boyko EJ, Asberry PJ, Hull RL, Kodama K, Callahan HS, Matthys CC, Leonetti DL, Schwartz RS, Kahn SE, Fujimoto WY: A reduced-fat diet and aerobic exercise in Japanese Americans with impaired glucose tolerance decreases intra-abdominal fat and improves insulin sensitività but not β-cell function. *Diabetes* 2005; 54:340–347
11. Mayer-Davis E, D'Agostino R Jr, Carter AJ, Haffner SM, Rewers MJ, Saad M, Bergman RN, IRAS Investigators: Intensity and amount of physical activity in relation to insulin sensitivity: The Insulin Resistance Atherosclerosis Study. *JAMA* 1998; 279:669-674
12. Hu G, Barengo NC, Tuomilehto J, Lakka TA, Nissinen A, Jousilahti P. Relationship of physical activity and body mass index to the risk of hypertension: a prospective study in Finland. *Hypertension* 2004; 43:25-30
13. Knowler WC, Barrett-Connor E, Fowler SE, Hamman RF, Lachin JM, Walker EA, Nathan DM; Diabetes Prevention Program Research Group: Reduction in the incidence of type 2 diabetes with lifestyle intervention or metformin. *N Engl J Med* 2002; 346:393-403
14. Hu G, Eriksson J, Barengo NC, Lakka TA, Valle TT, Nissinen A, Jousilahti P, Tuomilehto J: Occupational, commuting, and leisure-time physical activity in relation to total and cardiovascular mortality among Finnish subjects with type 2 diabetes. *Circulation* 2004; 110:666-673
15. Hu G, Jousilahti P, Barengo NC, Qiao Q, Lakka TA, Tuomilehto J: Physical activity, cardiovascular risk factors, and mortality among Finnish adults with diabetes. *Diabetes Care* 2005; 28:799-805
16. Alberti KG, Zimmet P, Shaw J; IDF Epidemiology Task Force Consensus Group. The metabolic syndrome - a new worldwide definition. *Lancet* 2005; 366(9491):1059-1062.
17. Apolone G, Mosconi P, The Italian SF-36 health Survey: Transation, validation and norming. J Clin Epidemiol 1998; 51: 1025-1036.
18. Nicolucci A, Giorgino R, Cucinotta D, Zoppini G, Muggeo M, Squatrito S, Corsi A, Lostia S, Pappalardo L, Benaduce E, Girelli A, Galeone F, Maldonato A, Perriello G, Pata P, Marra G, Coronel GA, Validation of the Italian version of the WHO-Well-Being Questionnaire (WHO-WBQ) and the WHO-Diabetes Treatment Satisfaction Questionnaire (WHO-DTSQ). Diabetes Nutr Metab 2004; 17: 235-243.
19. Di Loreto C, Fanelli C, Lucidi P, et al. Validation of a counseling strategy to promote the adoption and the maintenance of physical activity by type 2 diabetic subjects. *Diabetes Care* 2003; 26(2):404–408.
20. Folsom AR, Jacobs DR Jr, Caspersen CJ, Gomez-Marin O, Knudsen J. Test-retest reliability of the Minnesota leisure time physical activity questionnaire. *J Chron Dis* 1986; 39(7):505-511.
21. American College of Sports Medicine. ACSM's Guidelines for Exercise Testing and Prescription. 7^th^ Edition. Lippincott Williams & Wilkins, 2005.
22. Balke B, Ware R, An experimental study of physical fitness of Air Force personnel. *US Armed Forces Med J* 1959; 10: 675-688.
23. Brzycki M, Strength testing – predicting a one-rep max from reps to fatigue. *J Physical Education, Recreation & Dance* 1993; 64: 88-90.
24. Lakka TA, Venalainen JM, Rauramaa R, Salonen R, Tuomilehto J, Salonen JT. Relation of leisure-time physical activity and cardiorespiratory fitness to the risk of acute myocardial infarction in men. *N Engl J Med* 1994; 330:1549-1554
25. Ainsworth BE, Haskell WL, Leon DR, Jacobs HJ, Montoye HJ, Sallis JF, Paffenbarger RS. Compendium of physical activities: classifications of energy costs of human physical activities. *Med Sci Sports Exerc* 1993; 25: 71-80

**Appendix 1: IDES investigators**

**Diabetes Centers** (Figure 1)

1. Diabetes Division, S. Andrea Hospital and Department of Clinical Sciences, 2^nd^ Medical School, “La Sapienza” University, Rome: Francesco Fallucca MD; Giuseppe Pugliese MD, PhD; Serena Missori MD; Maria Cristina Ribaudo MD; Elena Alessi, MD
2. Division of Endocrinology and Metabolism, National Geriatric Institute (I.N.R.C.A.), Rome, Felice Strollo MD; Massimo Morè MD
3. Diabetes Unit, Villa S Pietro Hospital: Pietro Alimonti MD; Nicolina Di Biase MD; Filomena Lasaracina MD
4. Diabetes Unit, City Hospital of Civitavecchia: Graziano Santantonio MD
5. Diabetes Prevention Center (ACISMOM) Latina: Laura Cruciani MD
6. Diabetes Unit, “Triolo Zancla” Clinic, Palermo: Mario Manunta MD
7. Research Center for Physical Activity in Diabetes, University of Catania: Maurizio Di Mauro MD
8. Department of Experimental and Clinical Medicine. University of Catanzaro: Giorgio Sesti MD, Concetta Irace MD; and Diabetes Unit, City Hospital of Catanzaro: Luigi Puccio MD
9. Division of Endocrinology and Metabolism, University of Foggia: Mauro Cignarelli MD; Vincenzo Nicastro MD; Sabrina Piemontese MD
10. Diabetes Unit (AID), Provincial Health Authority for Naples1: Gerardo Corigliano MD; Ernesto Rossi MD; Marco Corigliano MD
11. Department of Internal Medicine,University of Perugia: Pierpaolo De Feo MD;Cristina Fatone MD
12. Diabetes Unit, New Hospital of San Giovanni di Dio, Florence: Cristiana Baggiore MD; Roberto Russo MD
13. Division of Diabetes and Metabolism, National Geriatric Institute (I.N.R.C.A), Ancona: Massimo Boemi MD; Luigi Lanari MD
14. Diabetes Unit, City Hospital of Brescia: Umberto Valentini MD; Angela Girelli MD
15. Diabetes Unit, City Hospital of Ravenna: Paolo Di Bartolo MD; Francesca Pellicano MD
16. Diabetes Unit, City Hospital of Rimini: Paolo Mazzuca MD
17. Diabetes Unit, City Hospital of Reggio Emilia: Enrica Manicardi MD
18. Diabetes Unit, San G. Battista Hospital, Turin: Alberto Bruno MD
19. Diabetes Unit, “Cà Foncello” Hospital, Treviso: Maria Sambataro MD
20. Diabetes Clinic, Monterotondo, Rome: Stefano Balducci MD
21. Department of Internal Medicine, San Paolo Hospital, University of Milan: Antonio Pontiroli MD; Marco Laneri MD; Anna Boggio MD
22. Diabetes Unit, Belcolle Hospital, Viterbo: Nunzio Zagari MD

**Metabolic Fitness Centers**

1. Center for Functional Assessment in Sport, Sant’Andrea Hospital, Rome: Fredrick Fernando, Carla Iacobini
2. Health Care Team, Monterotondo: Gianluca Balducci, Lorella Senigagliesi, Enza Spinelli
3. Athlos Club, Civitavecchia: Alessandro Di Giovanni
4. Metabolic Fitness, Latina: Mariano Pineda
5. DO-IN, Palermo: Umberto Pandolfo
6. The Wellness Center, Naples: Ciro Giordano
7. Elisir Club, Perugia: Antonella Settequattrini
8. Metagym, Florence: Marco Gambacciani
9. Fisioclub, Ancona: Matteo Fabrizi
10. Lifeplanet, Ravenna: Adriano Ceccherini, Enrico Balducci
11. Steven Sporting Club, Rimini: Mirco Quattrini
12. Centre for Physiatrics and Sports Medicine VITALIA, Torino: Massimo Massarini
13. Pianeta sport, Treviso: Giuseppe Baggio
14. Winner Clubs, Reggio Emilia: Marco Fornari
15. Millennium, Brescia: Davide Violi
16. GCube Fitness & Wellness, Viterbo: Giancarlo Cherubini
17. Zenith Centr for Medicine, Foggia: Fabio Mastelloni
18. S.S. Free Studios UISP, Catanzaro: Valeria Micali
19. Research Center for Physical Activity in Diabetes, Catania: Daniela Cilano, Simone Di Luciano
20. Centro la fonte del benessere, Milan: Davide Canevari

**Appendix 2: Informed consent**

# INFORMED CONSENT

**Title of study**: The Italian Diabetes Exercise Study (IDES)

**Principal investigator:** Stefano Balducci, MD, Contract Professor of Endocrinology

**Institution:** Diabetes Division, Sant’Andrea Hospital, “La Sapienza” University, Via di Grottarossa, 1035-1039 – 00189 Rome, Italy

## Introduction

## The Diabetes Division of Sant’Andrea Hospital, “La Sapienza” University, is conducting a research study to assess the efficacy of an intensive exercise intervention strategy in promoting physical activity and improving haemoglobin (Hb) A1c and other modifiable cardiovascular risk factors in patients with type 2 diabetes (T2DM) and the metabolic syndrome (MS). Since you are a patient suffering from these conditions, I would like to invite you to join this research study.

**Background information**

The U.S. Department of Health and Human Services and the American College of Sports Medicine recommend a minimum of 150 minutes/week of moderate-intensity exercise or, in moderately fit subjects, 60 minutes/week of vigorous activity. The American Diabetes Association has extended these prescriptions also to subjects with impaired glucose tolerance, to prevent development of T2DM, and to patients with T2DM, to improve glycaemic control, assist with weight maintenance, and reduce cardiovascular risk . However, it is debated whether the same volume of physical activity (PA) should be recommended to T2DM subjects, who have a higher cardiovascular risk than the general population. It is also essential to identify effective strategies to promote an adequate amount of PA in these patients. Counselling interventions have been recently designed and tested successfully in clinical settings and those focused only on exercise appear to be more effective than those targeting multiple behaviours. Moreover, meta-analyses indicated that supervised exercise is effective in improving cardio-respiratory fitness and glycaemic control. Finally, growing evidence suggests that resistance training is beneficial also in diabetic patients, and a recent trial showed that combined aerobic and resistance exercise is more effective than either one alone in ameliorating HbA1c. Therefore, resistance training is now recommended in diabetic patients in combination with aerobic exercise, or as an alternative when the latter is contraindicated, and also in subjects with the MS.

**Purpose of this research study**

The Italian Diabetes and Exercise Study (IDES) is aimed at assessing whether a strategy combining a prescribed and supervised mixed (aerobic and resistance) training program with structured exercise counselling is effective in promoting PA and improving HbA1c and other modifiable cardiovascular risk factors, in a large cohort of sedentary subjects with T2DM and the MS. This combined strategy will be compared to conventional disease management including exercise counselling, aimed at achieving the currently recommended volume of PA. The working hypothesis is that the combined strategy is superior to counselling alone in improving cardiovascular risk profile by allowing to attain a higher amount of PA.

**Procedures**

This study is enrolling sedentary patients with T2DM fulfilling the International Diabetes Federation criteria for the metabolic syndrome. Patients having any condition limiting or contraindicating exercise will be excluded from the study.

Patients eligible will be recruited and randomized to supervised aerobic and resistance training plus structured exercise counselling (exercise, EXE, group; n=300) versus counselling alone as part of standard care (control, CON, group; n=300) for 12 months. The allocation sequence will be generated by the co-ordinating centre and will be concealed until interventions are assigned. Standard care consists of a treatment regimen aimed at achieving optimal glycaemic, lipid, blood pressure (BP) and body weight targets, as established by current guidelines and including glucose-, lipid- and BP-lowering agents as needed and, when indicated, anti-platelet drugs. . For ethical reasons, drugs will be also adjusted throughout the study to maintain subjects at target levels and to account for reduced needs. This program includes also a dietary prescription, preceded by a preliminary nutritional evaluation with assessment of body mass index (BMI) and the individual patient requirements and preferences. Adherence to diet will be verified and dietary prescriptions will be adjusted at each intermediate visit

Both CON and EXE subjects will receive a structured individualized counselling intervention consisting of 7 steps, : motivation, self-efficacy, pleasure, support, comprehension, lack of impediments, diary. Counselling, which will be reinforced every 3 months, is aimed at achieving the currently recommended amount of PA by encouraging patients to perform commuting, occupational, home and leisure-time (LT) PA, without prescribing any specific type of activity.

The training program for the EXE group consists of 150 minutes/week in 2 supervised sessions of progressive mixed (aerobic and resistance) training. Aerobic training will be performed using treadmill, step, elliptical, arm or cycle-ergometer. Resistance training consists of 4 resistance exercises, i.e. thrust movement on the transverse plane (chest press or equivalent), traction movement on the frontal plane (lateral pull down or equivalent), squat movement (leg press or equivalent), trunk flexion for the abdominals, and three stretching positions. Intensity will be adjusted according to improvements in measures of cardio-respiratory fitness (predicted VO2max) and strength (1RM), respectively, which will be recorded throughout the study.

##### Possible risks or benefits

##### No significant side effects have been reported for this type of exercise.

##### There is no direct financial or other benefit for the participant of the study. However, all the investigations will be done free of cost to the patients. Treatment of any side effect will also be provided free of cost.

# **Confidentiality**

All information gathered from the study will remain confidential as per law 31dec 1996 n. 675. Your identity as a participant will not be disclosed to any unauthorized persons; only the researchers (the committee that approved this research project) will have access to the research materials. Any references to your identity that would compromise your anonymity will be removed or disguised prior to the preparation of the research reports and publications as per art. 13 law 675/96.

There will be no costs for participating in the research.

Participation in this study is voluntary; refusal to participate will not prejudice your medical care.

### Participation in the study

Any questions concerning the research project should be directed to your medical doctor, it is important that you read and sign the following page (Consent form) of this document. Your signature indicates that you have read and understood the above information, that you have discussed this study with the person obtaining consent, that you have decided to participate based on the information provided and that a copy of this form will be filed in the medical doctor archive responsible of the study and that a copy of this form has been given to you.

**INFORMED CONSENT FORM**

**TITLE OF RESEARCH:** Italian Diabetes Exercise Study

**AUTHORIZATION**

I have read and understood this consent form, and I volunteer to participate in this research study. I understand that I will receive a copy of this form. I voluntarily choose to participate, but I understand that my consent does not take away any legal rights in the case of negligence or other legal fault of anyone who is involved in this study. I further understand that nothing in this consent form is intended to replace any applicable state or local laws.

Participant’s Name (Printed or Typed):

Date:

Participant’s Signature or thumb impression:

Date:

Principal Investigator’s Signature:

Date:

Signature of Person Obtaining Consent:

Date:

**Appendix 3: Members of the Research Group Steering Committee**

**Coordinator:** *Giuseppe Pugliese MD PhD,* Diabetes Division, S. Andrea Hospital and Department of Clinical Sciences, 2^nd^ Medical School, “La Sapienza” University, Rome

**Statistics:** *Antonio* *Nicolucci MD,* Department of Clinical Pharmacology and Epidemiology, Consorzio Mario Negri Sud, S. Maria Imbaro CH, Italy

**Editing:** *Philippa Mungra,* University of Roma La Sapienza

**Questionnaires:** *Silvano Zanuso MSc,* Department of Motor Science, Faculty of Medicine, University of Padua, Padua, Italy

**Tutor Diabetologists**: *Stefano Balducci MD,* Metabolic Fitness Association

**Tutor Exercise Fitness**: *Gianluca Balducci, Paolo Benvenuti,* Metabolic Fitness Association

**Clinical Chemistry:** *Patrizia Cardelli MD, Stefano Cavallo MD,* Laboratory of Clinical Chemistry, S. Andrea Hospital, and Department of Cellular Biotechnology and Hematology, 2^nd^ Medical School, “La Sapienza” University, Rome

**Molecular Biology***: Maurizio Simmaco MD, Marina Borro, Giovanna Gentile,* Advanced Molecular Diagnostics, Sant’Andrea Hospital and Department of Biochemical Science**,** 2^nd^ Medical School, “La Sapienza” University, Rome

**Strength and VO2max evaluation**: *Massimo Massarini MD,* Vitalia Torino

**Arterial Hypertension**: *Claudio Letizia MD,* University of Roma La Sapienza

**Nontraditional CVD risk factors**: *Giuseppe Pugliese MD PhD,* Diabetes Division, S. Andrea Hospital and Department of Clinical Sciences, 2^nd^ Medical School, “La Sapienza” University, Rome

**Data and Safety Monitoring Board (DSMB)**: *Gruppo di studio Attività Fisica e Diabete di Diabete Italia*

**Figure 1:** Participating clinical centers


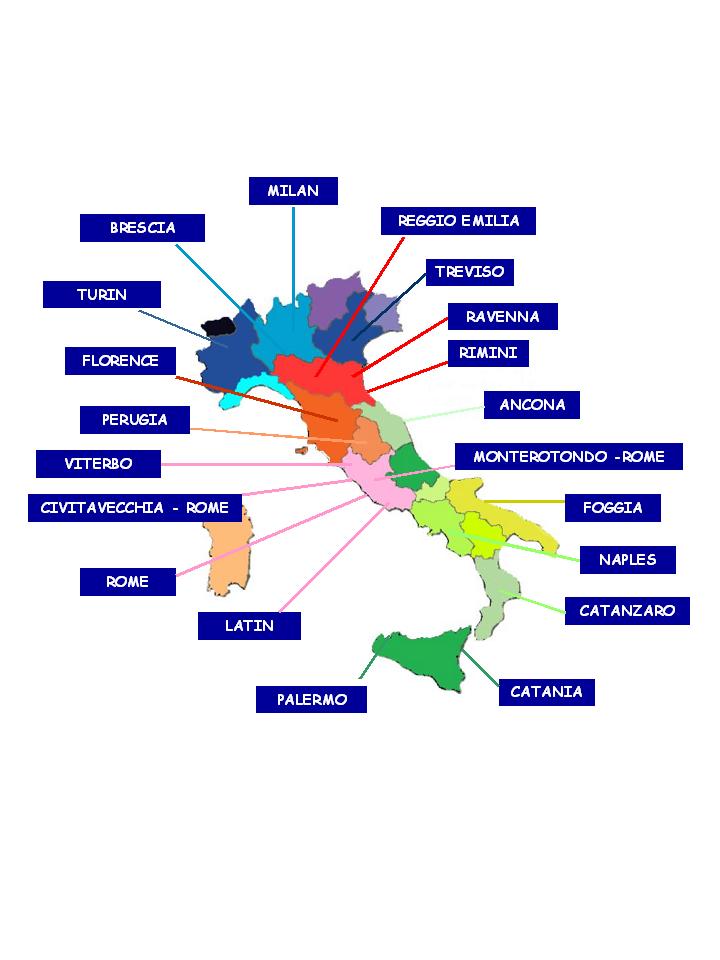


**Figure 2**: Cover and back cover (A) and example of a two-week page (B) of the Prospective Physical Activity diary utilized throughout the 12 months of the IDES intervention.

**A**

| 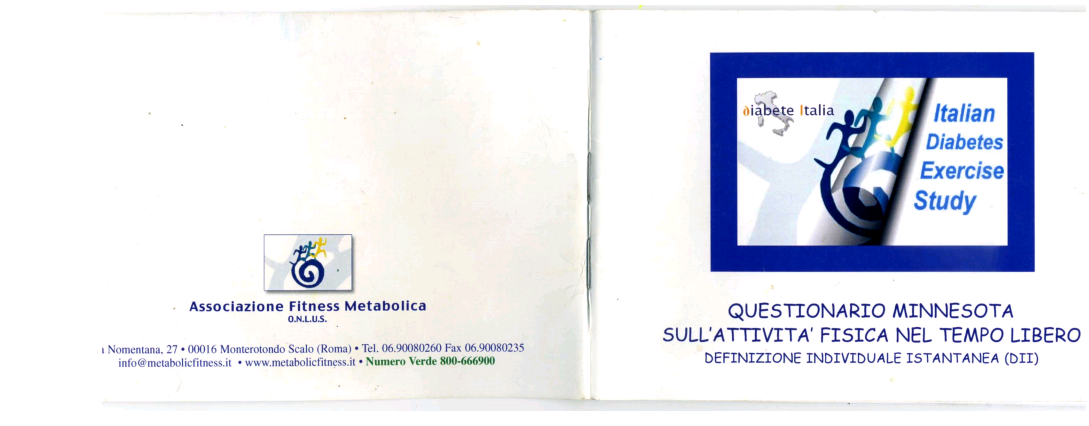 | 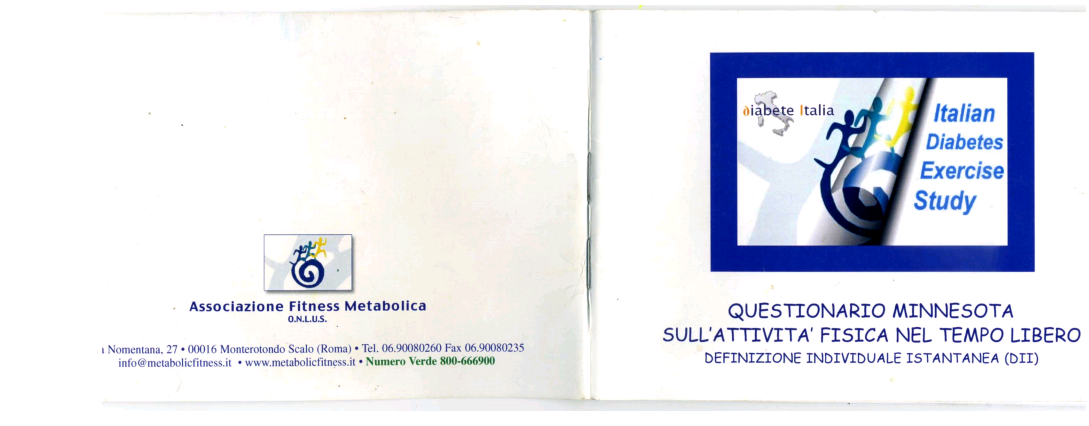 |
| --- | --- |

**B**

**Week Number Day of the Week Activity code Activity Duration Notes**


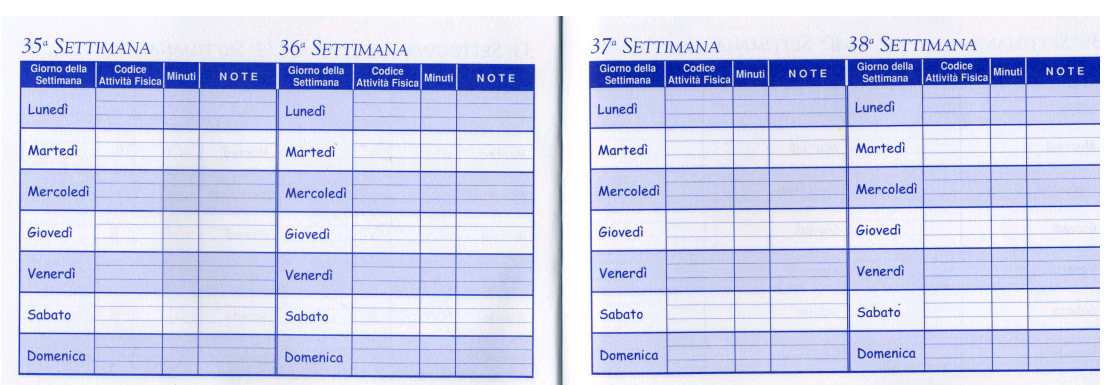


**Table 1:** Inclusion and exclusion criteria.

**Inclusion criteria**

1. Type 2 diabetic patients with Metabolic Syndrome, according to IDF criteria
2. Diabetes duration >1 year.
3. Age 40-75 years at screening
4. BMI 27- 40 kg/m^2^
5. Sedentary for at least 6 months*.
6. They must have been treated by prescribed diet and/or OHA e insulin
7. Able to walk for long distances unaided.
8. Eligible after positive outcome of cardiac evaluation .

* Sedentary patients were defined: (1) those expending less than 10% of their leisure time expenditure in activities involving > 4 MET; (2) those who did not practice any leisure time physical activity and who also were above the median in the number of hours spent sitting down during leisure time.

**Exclusion criteria**

1. All patients with a history of central nervous dysfunction such as hemiparesis, myelopathies, cerebral ataxia, significant musculoskeletal deformities such as an amputation, dysmetria, or scoliosis, patients with movement abnormalities or arthritis limited by pain when exercising.
2. A history of clinical evidence of severe cardiovascular disease which may limit or be a contraindication for exercise.
3. Clinical evidence of vestibular dysfunction
4. Angina and related symptoms
5. Postural hypotension defined as a fall in arterial blood pressure when changing position of >20 mmHg (systole) or >10 mmHg (diastole)
6. History of plantar sores.

**Table 2:** Recruitment and follow-up process

| **Phase** | **Visit** | **Action** |
| --- | --- | --- |
| **Recruitment** | **R1** | Registration of eligible patients in the IDES database together with their demographic, anthropometric and clinical data  Participation acceptance and informed consent sign  Prescription of ophthalmologist and cardiologist consultation for retinopathy and CVD evaluation |
|  | **R2** | Return of ophthalmologist and cardiologist evaluation  Administration of the SF-36 and the WHO-DTSQ questionnaires  Administration of the Minnesota LTPA questionnaire  Structured individualized counseling intervention  Collection of blood and urine samples for baseline, Group A biochemical measurements and forwarding to the central laboratory via express courier  Appointment at the Metabolic Fitness Center for metabolic fitness assessment and strength evaluation  Communication of the group assignment  Information about the exercise program (EXE group) |
|  | **R3** | Registration of results of baseline, group A biochemical measurements  Registration of data from Minnesota LTPA questionnaire  Nutritional evaluation and prescription of a treatment regimen according to the current guidelines, including diet prescription  Consignment of a diary for prospective assessment of physical activity  Prescription for intermediate, group B biochemical measurements |
| **Follow-up** | **F1** | Intermediate anthropometric and blood pressure measurements  Registration of results of intermediate, group B biochemical measurements (with eventual treatment regimen modification)  Registration of data from the diary of physical activity  Structured individualized counseling reinforcement  Prescription for intermediate, group B biochemical measurements |
|  | **F2** | Intermediate anthropometric and blood pressure measurements  Registration of results of intermediate, group B biochemical measurements (with eventual treatment regimen modification)  Registration of data from the diary of physical activity  Structured individualized counseling reinforcement  Prescription for intermediate, group B biochemical measurements |
|  | **F3** | Intermediate anthropometric and blood pressure measurements  Registration of results of intermediate, group B biochemical measurements (with eventual treatment regimen modification)  Registration of data from the diary of physical activity  Structured individualized counseling reinforcement |
|  | **F4** | Final anthropometric and blood pressure measurements  Collection of blood and urine samples for baseline, Group A biochemical measurements and forwarding to the central laboratory via express courier  Registration of data from the diary of physical activity  Administration of the SF-36 and the WHO-DTSQ questionnaires  Appointment at the Metabolic Fitness Center for metabolic fitness assessment and strength evaluation |

**Table 3:** Biochemical measurements

***Group A*** – to be performed at central laboratory (Laboratory of Clinical Chemistry, Sant’Andrea Hospital, Rome, Italy) at baseline and at the end of the study.

- HbA1c ^a^, blood glucose ^b^, C-peptide, and insulin levels ^c^ with calculation of the Homeostasis Model Assessment-Insulin Resistance (HOMA-IR) index from fasting glucose and insulin levels using the HOMA Calculator© software ([www.dtu.ox.ac.uk/homa/index.php](http://www.dtu.ox.ac.uk/homa/index.php));
- serum triglycerides and total and HDL-cholesterol levels ^b^ with LDL-cholesterol calculation using the formula: total cholesterol – [HDL-cholesterol + (triglycerides/5)];
- BUN, uric acid and serum creatinine ^b^, with calculation of estimated glomerular filtration rate (eGFR) is calculated from serum creatinine by the modified Modification of Diet in Renal Disease Study Group equation ([www.kidney.org/professionals/KDOQI/gfr_calculator.cfm](http://www.kidney.org/professionals/KDOQI/gfr_calculator.cfm)), and urinary albumin ^d^ / creatinine ^b^ ratio in spot urine sample (these data are presented elsewhere);
- serum aspartate aminotransferase, alanine aminotransferase, γ-glutamyl-transpeptidase and creatine phosphokinase levels ^b^;
- hs-C reactive protein ^b^.

^a^ High-performance liquid chromatography (Adams TMA1C HA-8160, Menarini Diagnostics, Florence, Italy).

^b^ VITROS 5,1 FS Chemistry System (Ortho-Clinical Diagnostics, Inc, Raritan, NJ, USA)

^c^ Chemiluminiscent immunometric assays (Immulite 2000 Thes; Diagnostic Products Corporation, Los Angeles, CA, USA)

^d^ mAlb VITROS (Ortho-Clinical Diagnostics, Inc)

***Group B***– to be performed in each centre laboratory at 3, 6 an 9 months.

- HbA1c and blood glucose levels;
- serum triglycerides and total and HDL cholesterol levels with LDL cholesterol calculation;
- BUN, serum creatinine, urinalysis, uric acid (if present hypeuricemia);
- serum aspartate aminotransferase, alanine aminotransferase, γ-glutamyl-transpeptidase and creatine phosphokinase levels (if treated with statins);
- plasma sodium and potassium levels (if treated with BP-lowering agents).

**Table 4:** List of Physical Activities (in Italian) and the relative METs intensities utilized to predict the accumulated volume of physical activity (METs·h^-1^·wk^-1^). Activities included and corresponding METS values are derived from the Minnesota Leisure Time Physical Activity Questionnaire.

| **PHYSICAL ACTIVITY** | **METS** |
| --- | --- |

1 Passeggiare 3.5

2 Camminare da casa al lavoro o nella pausa lavoro 4.0

3 Camminare (portando un carrello con la spesa) 3.5

4 Camminare (portando le sporte della spesa) 5.5

5 Salire le scale 8.0

6 Camminare in campagna/traking 6.07

7 Escursioni con lo zaino 7.0

8 Scalate in montagna 8.0

9 Andare in bicicletta al lavoro 4.0

10 Ballo 4.5

11 Aerobica o balletto 6.0

12 Giocare con i bambini 4.5

**Esercizi di mantenimento generale**

13 Ginnastica in casa 4.5

14 Ginnastica in palestra 6.0

15 Camminare velocemente 4.5

16 "Jogging" 6.0

17 Corsa 8-11 km/h 10.0

18 Corsa 12-16 km/h 15.0

19 Sollevamento pesi 6.0

**Attività acquatiche**

20 Sci acquatico 6.0

21 Surf 6.0

22 Navigazione a vela 3.0

23 Canotaggio o remi (dilettante) 3.5

24 Canotaggio o remi (professionista) 12.0

25 Fare un viaggio in canoa 4.0

26 Nuoto in piscina (piú di 150 metri) 6.0

27 Nuoto nel mare 6.0

28 Andare sott’acqua, snoorking 5.0

**Sport invernali**

29 Sci di discesa 7.0

30 Sci di fondo 8.0

31 Pattinaggio (ruote o ghiaccio) 7.0

**Altre attività**

32 Ippica 5.0

33 Boowling 3.0

34 Pallavolo 4.0

35 Ping-Pong 4.0

36 Tennis individuale 8.0

37 Tennis doppio 6.0

38 Badminton 7.0

39 Pallacanestro (non in partita) 6.0

40 Pallacanestro (giocando una partita) 8.0

41 Pallacanestro (da arbitro) 7.0

42 Squash 12.0

43 Calcio 10.0

44 Golf (portando il carrello) 3.5

45 Golf (camminando e portando le mazze) 5.5

46 Pallamano 10.0

47 Bocce 3.0

48 Arti marziali 10.0

49 Motociclismo 4.0

50 Ciclismo in strada o montagna 9.0

**Attività di giardinaggio**

51 Tagliare il prato con la falciatrice 4.5

52 Tagliare il prato manualmente 6.0

53 Pulire il giardino 4.5

54 Coltivare l’orto 5.0

55 Spalare la neve 6.0

**Lavori e attività casalinghe**

56 Lavoro di carpenteria in casa 3.0

57 Lavoro di carpenteria (all’aperto) 6.0

58 Imbiancare in casa 4.5

59 Imbiancare (all’aperto) 5.0

60 Pulire la casa 3.5

61 Spostare mobili 6.0

**Caccia e pesca**

62 Tiro con la pistola 2.5

63 Tiro con l’arco 3.5

64 Pesca in riva al mare 3.5

65 Pesca nel fiume (con gli stivali dentro l’acqua) 6.0

66 Caccia piccola 5.0

67 Caccia grossa (cervi, orsi...) 6.0

**Altro (specificare)**

68 .…………………………………………

69 .…………………………………………

70 .…………………………………………

**Table 5:** Progressive increase in global caloric expenditure per session.

| **1^st^ month** | **3.0 Kcal/Kg BW** |
| --- | --- |
| **2^nd^ month** | **3.1 Kcal/Kg BW** |
| **3^th^month** | **3.2 Kcal/Kg BW** |
| **4^th^ month** | **3.3 Kcal/Kg BW** |
| **5^th^ month** | **3.4 Kcal/Kg BW** |
| **6^th^ month** | **3.5 Kcal/Kg BW** |
| **7^th^ month** | **3.6 Kcal/Kg BW** |
| **8^th^ month** | **3.7 Kcal/Kg BW** |
| **9^th^ month** | **3.8 Kcal/Kg BW** |
| **10^th^ month** | **3.9 Kcal/Kg BW** |
| **11^th^ month** | **4.0 Kcal/Kg BW** |
| **12^th^ month** | **4.1 Kcal/Kg BW** |

**Table 6:** Modified Balke protocol.

| **Step** | **Duration** | **Speed** | **Gradient** |
| --- | --- | --- | --- |
| 1 | 1 min. | 4,3 Km/h | 0 % |
| 2 | 1 min. | 4,3 Km/h | 2 % |
| 3 | 1 min. | 4,3 Km/h | 3 % |
| 4 | 1 min. | 4,3 Km/h | 4 % |
| 5 | 1 min. | 4,3 Km/h | 5 % |
| 6 | 1 min. | 4,3 Km/h | 6 % |
| 7 | 1 min. | 4,3 Km/h | 7 % |
| 8 | 1 min. | 4,3 Km/h | 8 % |
| 9 | 1 min. | 4,3 Km/h | 9 % |
| 10 | 1 min. | 4,3 Km/h | 10 % |
| 11 | 1 min. | 4,3 Km/h | 11 % |
| 12 | 1 min. | 4,3 Km/h | 12 % |
| 13 | 1 min. | 4,3 Km/h | 13 % |
| 14 | 1 min. | 4,3 Km/h | 14 % |
| 15 | 1 min. | 4,3 Km/h | 15 % |
| 16 | 1 min. | 4,3 Km/h | 16 % |
| 17 | 1 min. | 4,3 Km/h | 17 % |
| 18 | 1 min. | 4,3 Km/h | 18 % |

**Table 7:** ACSM’s equations for calculation of energy expenditure during aerobic exercise.

**Treadmill**

Walking speed: VO_2_ = [(speed(km/h)*1000 / 60*0,1)+(slope / 100*speed*1000 / 60*1,8)+3,5]

Running speed: VO_2_ = [(speed(km/h)*1000 / 60*0,2)+(slope / 100*speed*1000 / 60*1,8*0,5)+3,5]

**Ergometer**

Cycle: VO_2_ = [(power (watt) * 6,12 *1,8) / body weight (kg) +7]

Arm: VO_2_ = [(power (watt) * 6 * 3) / body weight (kg) + 3.5]

Elliptical: VO_2_ = A + B * power (watt) + C * power (watt)^2^ + D * power (watt) + E * power (watt) ^2^

body weight (kg) body weight (kg)

+ F * watt + G * power (watt)

body weight (kg)

*Coefficients:*

A =   3.5 ( basal  VO_2_)

B =    -0.05544

C =   +0.00024

D =   +7.06881

E =    -0.00612

F =    +0.29396

G =    -1.31488

**Step**

VO_2_ = 7 + 0.257 * SPM (steps per minute)
