# Supplementary material for: Effect of High- versus Low-Intensity Supervised Aerobic and Resistance Training on Modifiable Cardiovascular Risk Factors in Type 2 Diabetes; The Italian Diabetes and Exercise Study (IDES)
Source: PLoS One. 2012 Nov 21;7(11):e49297. doi: 10.1371/journal.pone.0049297 (PMC3504024; doi:10.1371/journal.pone.0049297)
Supplement: Table S1 — Supplemental Table 1. (DOC) [file pone.0049297.s004.doc]

**Supplemental Table 1.** Additional baseline information.

|  | **LI** | **LI** |
| --- | --- | --- |
| **Sex, males/females** | 86/50 | 88/64 |
| **Age, years** | 58.4 (8.9) | 59.5 (8.3) |
| **Smoking habits, n (%)** |  |  |
| **Never** | 86 (63.3) | 93 (61.2) |
| **Former** | 21 (15.4) | 33 (21.7) |
| **Current** | 29 (21.3) | 26 (17.1) |
| **Diabetes duration, years** | 5.9 (4.0) | 7.8 (6.2) |
| **Family history of diabetes, n (%)** | 79 (58.1) | 110 (72.4) |
| **Diabetic complications, n (%)** |  |  |
| **Retinopathy** | 18 (13.2) | 27 (17.8) |
| **Nefrophathy** | 30 (22.1) | 44 (28.9) |
| **Acute myocardial infarction** | 7 (5.3) | 9 (5.8) |
| **Stroke** | 0 (0.0) | 0 (0.0) |
| **Foot ulcer** | 1 (0.7) | 11 (7.2) |
| **Revascularization** | 5 (3.7) | 8 (5.3) |

Values are mean (SD), unless otherwise noted; LI = low-intensity subgroup; HI = high-intensity subgroup.
